# Whole-brain millisecond-scale effective connectivity of auditory and visual naming

**DOI:** 10.1016/j.neuroimage.2026.122236

**Published:** 2026-09-14

**Authors:** Ryuzaburo Kochi, Aya Kanno, Hiroshi Uda, Keisuke Hatano, Masaki Sonoda, Hidenori Endo, Michael Cools, Robert Rothermel, Aimee F. Luat, Eishi Asano

**Affiliations:** a Department of Pediatrics, Children’s Hospital of Michigan, Wayne State University, Detroit Medical Center, Detroit, MI, 48201, USA; b Department of Neurosurgery, Tohoku University, Sendai, 9808575, Japan; c Department of Neurosurgery, Sapporo Medical University, Sapporo, Hokkaido, 0608543, Japan; d Department of Neurosurgery, Osaka Metropolitan University Graduate School of Medicine, Osaka, 5458585, Japan; e Department of Neurosurgery, Yokohama City University Graduate School of Medicine, Yokohama, 2360004, Japan; f Department of Neurosurgery, Children’s Hospital of Michigan, Detroit Medical Center, Detroit, MI, 48201, USA; g Department of Psychiatry, Children’s Hospital of Michigan, Wayne State University, Detroit Medical Center, Detroit, MI, 48201, USA; h Department of Pediatrics, Central Michigan University, Mt. Pleasant, MI, 48858, USA; i Department of Neurology, Children’s Hospital of Michigan, Wayne State University, Detroit Medical Center, Detroit, MI, 48201, USA

**Keywords:** Epilepsy surgery, Language organization, Dynamic causal tractography atlas

## Abstract

**Objective:**

Clinical mapping studies in epilepsy and brain tumor surgery have informed neurobiological models of speech. However, a comprehensive model requires clarification of when, for how long, and in what directions cortical regions transmit information to support processes ranging from perception and comprehension to lexical retrieval and articulation. We aimed to characterize whole-brain patterns of directional neural information flow during auditory and visual naming.

**Methods:**

We studied 127 patients who performed auditory and picture naming tasks during intracranial EEG recordings, analyzing 9526 artifact-free nonepileptic recording sites. We applied transfer entropy–based effective connectivity analysis to estimate information flow between brain regions. Information flow was classified as *accelerating* when increases in group-level high-gamma amplitude in one region predicted subsequent increases in another region connected by white matter, and *decelerating* when decreases predicted subsequent decreases in the downstream region. The functional relevance of these information flow patterns was evaluated using electrical stimulation mapping and behavioral response times.

**Results:**

Following stimulus onset, accelerating flows emerged from modality-specific sensory cortices and propagated to higher-order regions before becoming bidirectional. These transient accelerating flows were typically followed by decelerating phases, during which new accelerating flows arose. Stronger accelerating flows at specific time points predicted a higher probability of stimulation-induced symptoms (Spearman’s rho: 0.54–0.81; *p* < 0.00,001). During auditory naming, faster responses were associated with stronger accelerating flows within left perisylvian pathways, whereas during visual naming faster responses were associated with stronger accelerating flows within bilateral basal temporal pathways.

**Discussion:**

Strong accelerating flows, followed rapidly by decelerating flows, predicted stimulation-induced symptoms and faster behavioral responses, indicating that information transfer through white matter supports sequential transitions from perception to lexical retrieval and articulation. By resolving the direction of neural propagation through white matter pathways, this study provides a whole-brain model of temporally precise, anatomically specific, and directional network interactions underlying human speech.

## 1. Introduction

Speech arises from dynamic neural interactions among distributed cortical regions mediated by long-range white matter pathways (Hickok and Poeppel, 2007; Dick and Tremblay, 2012; Chang et al., 2015; Hagoort, 2019; Gajardo-Vidal et al., 2021). Electrical stimulation mapping can determine the causal necessity of specific cortical regions (Tate et al., 2014; Nakai et al., 2017; Lu et al., 2021; Woolnough et al., 2021; Perrone-Bertolotti et al., 2020). However, characterizing the spatiotemporal organization of speech at the whole-brain level remains challenging because it requires approaches capable of resolving directional neural information flow while incorporating structural connectivity. Our prior intracranial EEG (iEEG) studies demonstrated that structurally connected cortical regions coactivate during speech, and that the timing and magnitude of coactivation predict stimulation-induced symptoms (Kanno et al., 2025; Kochi et al., 2026). Yet a fundamental question remains unanswered: when, for how long, and in which directions does neural information flow across distributed cortical networks during speech, and how do these flows contribute to speech generation?

To address this question, we analyzed iEEG recordings obtained during auditory and visual naming to resolve the timing, directionality, and white matter pathways underlying neural information flow. By linking temporally resolved flow dynamics to electrical stimulation outcomes and behavioral response times, we delineated distinct phases of modality-specific speech processing—including perception, semantic access, lexical retrieval, response planning, and articulatory execution. Because perceived speech stimuli need to be transmitted from sensory cortices to downstream conceptual and motor systems (Binder, 2017; Wurm and Caramazza, 2022), we hypothesized that unidirectional information flow from modality-specific sensory regions to higher-order areas would emerge early in processing. Consistent with prior demonstrations of later-stage interactions within perisylvian frontotemporal and occipitotemporal circuits (Woolnough et al., 2021; Thesen et al., 2012; Flinker et al., 2015), we further predicted that white matter pathways linking these regions (Gajardo-Vidal et al., 2021; Conroy et al., 2018) —particularly the arcuate fasciculus and inferior longitudinal fasciculus—would mediate semantic access and lexical retrieval.

Here, we quantified neural information flow using transfer entropy-based effective connectivity analysis (Schreiber, 2000; Ito et al., 2011; Firestone et al., 2025). Flows were classified as *accelerating* when increasing high-gamma amplitude (70–110 Hz) in one region predicted subsequent increases in a structurally connected region, and as *decelerating* when decreases predicted downstream decreases. High-gamma activity was selected because it is closely correlates with neuronal firing (Ray et al., 2008), metabolic demand (Nishida et al., 2008), and hemodynamic activation (Nir et al., 2007). High-gamma activity provided millisecond-scale temporal resolution and greater sensitivity for predicting postoperative cognitive outcomes than lower-frequency bands (Sonoda et al., 2022; Ervin et al., 2025). Together, this large-cohort study integrates whole-brain visualization of directional neural information flow through white matter pathways with stimulation-based measures of causal relevance and behavioral performance.

## 2. Materials and methods

### 2.1. Participants

From January 2007 through December 2024, we recruited individuals with focal seizures who fulfilled the eligibility criteria (Kochi et al., 2026). Eligible participants were native English speakers, four years of age or older, who completed auditory or visual naming tasks during extraoperative iEEG at the Detroit Medical Center (eFigure 1). We excluded those with sensory deficits, a history of epilepsy surgery, major malformations, or evidence of right-hemisphere language dominance, determined by Wada testing or by the combination of left-handedness and congenital left-hemispheric cortical lesions. With these criteria, the left hemisphere was assumed to contain essential language functions (Sonoda et al., 2022; Möddel et al., 2009).

### 2.2. Intracranial electrodes

Intracranial implantation of platinum disk or depth electrodes was performed (Kochi et al., 2026; Sonoda et al., 2022). A cortical surface reconstruction was generated, and electrode locations were determined by registering the post-implant CT scan to the preoperative MRI using FreeSurfer (http://surfer.nmr.mgh.harvard.edu) (Desikan et al., 2006). Electrode coordinates were transformed into the FreeSurfer standard space and mapped to predefined regions of interest (ROIs) (eFigure 2) (Sakakura et al., 2023).

### 2.3. Structural connectivity

We assessed structural connectivity between ROIs using tractography data from the Human Connectome Project (http://brain.labsolver.org/diffusion-mri-templates/hcp-842-hcp-1021) (Yeh et al., 2018). This approach is appropriate, as the goal was to model neural interactions representative of broader populations rather than tailored to a single patient (Kochi et al., 2026; Sakakura et al., 2023). We demonstrated that white-matter trajectories in children with epilepsy are consistent with those in healthy individuals (Sonoda et al., 2021). White-matter connections linking ROIs were visualized in Montreal Neurological Institute space using DSI Studio (http://dsi-studio.labsolver.org/). Parameters for tract reconstruction are detailed previously, and the corresponding white-matter imaging dataset is publicly available (Kochi et al., 2026).

### 2.4. iEEG

Patients were monitored with video-iEEG with signals digitized at 1000 Hz (Kochi et al., 2026). Analyses were restricted to artifact-free electrodes at nonepileptic electrode sites, clinically and operationally defined as those outside seizure onset, interictal epileptiform discharges, or MRI-visible lesions (Sakakura et al., 2023).

### 2.5. Tasks

Participants completed auditory and visual naming paradigms (Kochi et al., 2026; Sonoda et al., 2022). For the auditory condition, up to 100 spoken questions (e.g., “What flies in the sky?”) required brief noun responses. Stimulus and response onsets were tracked with a photosensor and microphone, and response latency was calculated from the end of the auditory cue to the response onset. In the visual condition, up to 60 grayscale pictures (e.g., “dog”) were presented on a screen for overt naming, with response latency defined from stimulus onset to response onset. Trials lacking valid responses were excluded from analysis.

### 2.6. Time-frequency analysis

Analysis was performed as previously described (Nakai et al., 2017; Kochi et al., 2026). High-gamma amplitude in the 70–110 Hz range was extracted using a bipolar montage between adjacent electrode contacts. Time–frequency decomposition was conducted using a Gabor transform with 5-ms temporal resolution and 10-Hz frequency bins in BESA Software (BESA, Gräfelfing, Germany) (Hoechstetter et al., 2004). Amplitudes at each time–frequency bin were converted to percent change relative to a 400-ms baseline period (−600 to −200 ms before stimulus onset). At each 5-ms time point, percent change in amplitude (defined as the square root of power) was calculated for each 10-Hz bin, and high-gamma amplitude was defined as the mean percent change across the 70–110 Hz range. Thus, each 10-Hz frequency bin contributed equally to the final high-gamma measure. iEEG recordings were aligned to stimulus onset, stimulus offset, and response onset during auditory naming, and to stimulus and response onsets during visual naming. Analysis windows covered −200 to +500 ms around stimulus onset and ±500 ms around stimulus offset and response onset. The spatial distribution of high-gamma modulation was rendered on a FreeSurfer cortical surface with 10-mm interpolation.

### 2.7. Group-level assessment of high-gamma amplitudes

We computed group-level mean high-gamma amplitudes for 66 ROIs, each based on data from ≥7 patients (eTable 2) (Solomon et al., 2017). This framework enables modeling of population-level neural interactions (Burke et al., 2014), analogous to cross-sectional or meta-analytic designs when longitudinal data or comprehensive sampling within a single cohort are not feasible (Binder et al., 2025). We assessed the robustness of group-level high-gamma amplitudes by illustrating temporal dynamics with confidence intervals and applying a leave-one-out validation approach (eFigures 3-6).

### 2.8. Accelerating and decelerating flows

Using transfer entropy-based effective connectivity analysis (Schreiber, 2000; Ito et al., 2011; Firestone et al., 2025), we quantified the strength and direction of neural information flow along white matter pathways. Methodological implementation details are available in our publicly accessible analysis code. Transfer entropy was applied to binarized high-gamma state transitions treated as stochastic processes. Specifically, transfer entropy was computed within 100-ms windows, each comprising twenty 5-ms time bins. Each bin was coded as 1 or 0. A bin was coded as 1 when high-gamma amplitude was significantly greater than the mean of the immediately preceding ten bins, as determined by *t*-tests sustained for five consecutive bins; otherwise, it was coded as 0. Across ROIs, high-gamma acceleration events occurred at multiple time bins (eFigures 3-4), generating informative windows with non-zero state transitions for count-based probability estimation. This binary time series served as input to the ‘Delayed Transfer Entropy’ algorithm (Ito et al., 2011) to estimate directional information flow between ROI pairs.

$TE_{ROI_{1}\to ROI_{2}}=\sum p(y_{t+1},y_{t},x_{t+1-d})\log_{2}\frac{p(y_{t+1}|y_{t},x_{t+1-d})}{p(y_{t+1}|y_{t})}$where $y_{t+1}$ denotes the binary state of ROI_2_ at time t+1, $y_{t}$ denotes the previous binary state of ROI_2_, $x_{t+1\text{-}d}$ denotes the binary state of ROI_1_ occurring d time bins before t + 1, p denotes probability, and d denotes the delay parameter. In the present study, transfer entropy was computed across delay parameters of *d* = 1, 2, and 3 bins (corresponding to 5, 10, and 15 ms), and the largest transfer entropy value (TEPk) was used to define neural information flow, consistent with the delayed transfer entropy approach described by Ito et al. (2011) (Ito et al., 2011). Transfer entropy quantifies the extent to which knowledge of the preceding state of ROI_1_ improves prediction of the future state of ROI_2_ beyond the information contained in ROI_2_ itself. Positive transfer entropy values were interpreted as evidence of directed statistical dependence from ROI_1_ to ROI_2_, consistent with neural information flow taking place from ROI_1_ to ROI_2_ through a direct white matter pathway. *Accelerating* flow from ROI_1_ to ROI_2_ was defined when ROI_1_ and ROI_2_ were connected by a white matter tract and transfer entropy was greater than zero, such that increasing high-gamma amplitude at ROI_1_ predicted a subsequent increase at ROI_2_. The transfer entropy value was interpreted as efferent flow at ROI_1_ and afferent flow at ROI_2_. The 100-ms analysis window slid in 5-ms increments. For visualization, flow strength at each 5-ms bin was estimated using a Gaussian-weighted summation of transfer entropy values within the corresponding window. *Decelerating* flow was defined analogously, when decreasing high-gamma amplitude at ROI_1_ predicted a subsequent decrease at ROI_2_.

We hypothesized that, during auditory naming, *accelerating* flow would initially be unidirectional from the left posterior superior temporal gyrus (pSTG) to the posterior middle temporal gyrus (pMTG) and subsequently become bidirectional. We similarly hypothesized that, during visual naming, *accelerating* flow would begin as unidirectional from the lateral occipital gyrus to the posterior fusiform gyrus and later evolve into bidirectional interactions within each hemisphere.

### 2.9. Functional coactivation and co-deactivation

Using the previously reported method (Kochi et al., 2026), we analyzed temporal windows and white matter pathways exhibiting functional coactivation or co-deactivation. Coactivation and co-deactivation quantify neural interactions along white matter pathways but do not provide information regarding directionality. In brief, ROI pairs were classified as coactivated (or co-deactivated) when both regions simultaneously demonstrated significant high-gamma augmentation (or attenuation) relative to the prestimulus baseline and the regions were directly connected by a white matter tract.

### 2.10. Effect of patient demographics and clinical factors on behavioral and neural measures

As previously reported (Kochi et al., 2026), mixed-effects models were used to examine whether age (√years), left-handedness, left--hemispheric epileptogenic focus, or anti-seizure medications were associated with response times or high-gamma augmentation (eResults).

### 2.11. Electrical stimulation

Electrical stimulation mapping was used to determine the functional relevance of *accelerating* flows. Full methodological descriptions are provided elsewhere (Kanno et al., 2025; Kochi et al., 2026; Sonoda et al., 2022). During 50-Hz stimulation of neighboring electrode pairs, patients were asked to answer *wh*-questions or name objects, and blinded examiners confirmed reproducible responses. Analyzed manifestations included auditory hallucinations, receptive and expressive aphasia, speech arrest, facial sensorimotor phenomena, phosphenes, visual distortions, and visual-naming errors.

Electrical stimulation mapping was performed in all patients. We attempted to stimulate all electrodes whenever feasible; however, because of time constraints, not all electrodes were stimulated in every patient. Consequently, the probability of stimulation-induced symptoms at each ROI was computed from the electrodes that underwent stimulation mapping.

### 2.12. Association between neural dynamics and stimulation-induced manifestations

We examined whether the intensity of *accelerating* flow at specific time bins, quantified by transfer entropy, was associated with the probability of given stimulation-induced symptoms. For each cortical ROI and each time bin, mean efferent flow intensity was computed by averaging transfer entropy values across all outgoing ROI pairs. Spearman’s rank correlation tested whether higher flow intensity at a cortical ROI was associated with a greater probability (%) of specific symptoms. Statistical significance was defined as a Bonferroni-corrected, two-sided *p* < 0.05 across 500 time bins. A positive correlation indicated that cortical ROIs exhibiting stronger neural information flow at a given time point were more likely to be associated with a given stimulation-induced symptom; thus, the spatial distribution of neural information flow across cortical ROIs corresponded to the spatial distribution of stimulation-induced manifestations. The same analysis was performed to evaluate the association between mean afferent flow intensity and stimulation-induced manifestations.

### 2.13. Neural correlates of within-individual variability in response times

We examined whether neural information flow within each time window was functionally relevant by testing its association with response times, while controlling for inter-patient variability by stratifying each patient's trials into six response time categories. For each category, we computed the mean flow intensity across all ROI pairs and assessed its association with response times using Spearman’s rank correlation. Statistical significance was defined as a Spearman’s rank correlation with *p* < 0.05 that was sustained across 10 consecutive 5-ms bins (i.e., a 50-ms period). We did not apply a conventional multiple-comparison correction across the 500 analyzed time bins. Instead, we adopted a temporal-clustering criterion requiring significance to persist across 10 consecutive bins. This approach was intended to reduce the likelihood of identifying isolated spurious correlations attributable to multiple testing while preserving sensitivity to temporally sustained neural effects. Our primary objective was to identify neural information flows predictive of subsequent naming performance. We hypothesized that stronger accelerating flow in cortical regions connected by the left arcuate fasciculus and the bilateral inferior longitudinal fasciculi would be associated with shorter response times during auditory and visual naming tasks, respectively.

### 2.14. Ethics statement

This study was approved by the Wayne State University Institutional Review Board (IRB No 048404MP2E). Written informed consent was obtained from all participants or their legal guardians, and assent was obtained from children aged 13 years or older, as appropriate.

### 2.15. Data availability

The data are available: https://openneuro.org/datasets/ds006910/versions/1.0.1 https://openneuro.org/datasets/ds006914/versions/1.0.3

### 2.16. Code availability

The codes are available: https://github.com/rkwsu/Project_Auditory_Visual_EC

## 3. Results

### 3.1. Impact of patient profiles on response time and neural measures

We found 127 eligible patients (age 5–49 years; 61 females; eTable 1); 121 completed the auditory task, 110 the visual task, and 104 completed both. We analyzed 9526 artifact-free nonepileptic sites (9178 for auditory, 8585 for visual naming; eTable 2).

The median response times, averaged across patients, were 1.46 and 1.38 s for auditory and visual naming, respectively (eTable 1). Older age was independently associated with faster median response times but not with high-gamma amplitude after adjustment for other factors. In contrast, left-handedness and a left-hemispheric epileptogenic focus were associated with reduced left-hemispheric dominance of high-gamma augmentation, independent of other covariates (eResults).

### 3.2. Robustness of group-level high-gamma amplitudes

Spearman correlation analyses demonstrated strong consistency in mean high-gamma amplitudes across datasets each excluding one patient (rho≥0.95; *p* < 0.00,001; eFigures 5-6).

### 3.3. Overview of neural interactions during naming

Neural information flows during auditory and visual naming are presented with functional coactivation/co-deactivation (eVideos 1-2) and cortical high-gamma modulation (eVideos 3-4). Accelerating flow initially propagated from modality-specific sensory cortices to higher-order regions, later becoming bidirectional and coinciding with sustained functional coactivation. Accelerating flows were transient (<500 ms) and followed by decelerating flows; in contrast, functional coactivation persisted and slowly subsided.

During auditory naming, bidirectional accelerating flows were most prominent along the left arcuate fasciculus, whereas during visual naming, they were distributed more evenly across multiple tracts, including the bilateral inferior longitudinal, left arcuate, and left inferior fronto-occipital fasciculi. Additional fasciculi also exhibited accelerating flows concurrently. eFigures 7-10 depict the proportion of each fasciculus demonstrating accelerating and decelerating flows across time bins, and eFigures 11-14 depict the proportion exhibiting functional coactivation and co-deactivation. These **eFigures** assist readers in appreciating that accelerating flows are transient, whereas functional coactivation persists over a longer period.

### 3.4. Auditory naming

Following stimulus onset, accelerating flow propagated from the pSTG to adjacent gyri before becoming bidirectional (eVideo 1; Fig. 1A). Unidirectional accelerating flow emerged from the left pSTG to the pMTG via U-fibers, peaking at 45 ms (Fig. 2A). Around 200 ms, bidirectional accelerating flow between the left pSTG and pMTG was observed. Concurrently, bidirectional accelerating flow occurred between the right and left pSTG via callosal fibers.

Peaking at 55 ms after stimulus onset, unidirectional accelerating flow emerged from the pSTG to the inferior precentral gyrus via the left arcuate fasciculus (Fig. 2B). Around 180 ms, accelerating flow from the left inferior precentral gyrus to the pSTG was observed.

Peaking 100–300 ms before stimulus offset, bidirectional accelerating flows were evident between the lateral temporal and perisylvian frontal regions via the left arcuate fasciculus (Figs. 1B-1C). Fig. 2C illustrates the dynamics of bidirectional flows between the left pMTG and the pars opercularis of the inferior frontal gyrus (IFG) during this period.

Around stimulus offset, bidirectional accelerating flows through the left arcuate fasciculus subsided (eVideo 1), whereas bidirectional accelerating flows across the left frontal lobe became prominent via the frontal aslant fasciculus and U-fibers. Fig. 2D shows the dynamics of bidirectional accelerating flows between the pars opercularis of the left IFG and the posterior superior frontal gyrus (pSFG). Peaking 200–300 ms after stimulus offset, bidirectional accelerating flows became evident between the left pSFG and the inferior precentral gyrus (Fig. 2E).

Around response onset, bidirectional accelerating flows were confined to intra- and interhemispheric pathways involving the Rolandic cortices and pSTG (Fig. 1E).

### 3.5. Auditory naming-related flows and stimulation-induced manifestations

eFigure 15 shows probability maps of stimulation-induced symptoms, while Fig. 3 illustrates how correlations between efferent flow intensity and symptom probability evolve over time. Peak correlations occurred at different time points:

- Auditory hallucination: 5 ms post-stimulus onset (rho: +0.58; *p*-value: 4.7 × 10^−7^)
- Receptive aphasia: 165 ms pre-stimulus offset (rho: +0.60; *p*-value: 1.5 × 10^−7^)
- Expressive aphasia: 80 ms pre-stimulus offset (rho: +0.81; *p*-value: 2.1 × 10^−16^)
- Speech arrest: 330 ms post-stimulus offset (rho: +0.69; *p*-value: 1.4 × 10^−10^)
- Facial sensorimotor symptom: 95 ms post-response onset (rho: +0.60; *p*-value: 1.1 × 10^−7^).

Afferent and efferent flow intensities demonstrated similar patterns of correlation with symptom probability (eFigure 16).

### 3.6. Neural correlates of variability in response times in auditory naming

Fig. 4 illustrates the timing and location of significant correlations between neural information flow intensity and response time. Around 400 ms after stimulus onset, stronger afferent decelerating flow to the right pSTG was associated with slower responses (maximum rho: +1.00; *p*-value: 0.003; Fig. 4A). Around 250 ms before stimulus offset, stronger efferent and afferent accelerating flows at the left pMTG and inferior precentral gyrus were associated with faster responses (minimum rho: −0.94; *p*-value: 0.016; Fig. 4B); these cortical regions are connected through the arcuate fasciculus.

### 3.7. Visual naming

After stimulus onset, accelerating flow propagated from the lateral occipital gyrus to the posterior fusiform gyrus in each hemisphere before becoming bidirectional and coinciding with sustained functional coactivation (eVideo 2; Figs. 5A-5B). Fig. 6A illustrates unidirectional intra-hemispheric accelerating flows emerging from the left lateral occipital to posterior fusiform gyrus via the inferior longitudinal fasciculus, peaking at 65 ms post-stimulus onset. Subsequently, accelerating flows from the left posterior fusiform to lateral occipital gyrus appeared, with peak latency of 400 ms (Fig. 6A). Concurrently, bidirectional accelerating flows were observed between these posterior cortical regions via callosal fibers.

Between 350 and 450 ms after stimulus onset, bidirectional accelerating flows also took place between the lateral occipital and posterior inferior temporal gyrus (pITG), between the left lateral occipital gyrus and inferior precentral gyrus via the superior longitudinal fasciculus, and between the left pITG and inferior precentral gyrus via the arcuate fasciculus (Figs. 5B, 6B, and 6C).

Between 350 and 450 ms prior to response onset, bidirectional accelerating flows were observed across the inferior precentral gyrus, pars opercularis of the IFG, and pSFG in the left hemisphere (Figs. 5C, 6D, and 6E).

During responses, intra- and inter-hemispheric accelerating flows were confined to the Rolandic areas, whereas functional coactivation continued to encompass posterior head regions (Fig. 5D).

### 3.8. Visual naming-related flows and stimulation-induced manifestations

Correlations between efferent accelerating flow and stimulation-induced manifestations peaked at distinct times (Fig. 7):

- Phosphenes at 90 ms post-stimulus (rho: +0.75; *p*-value: 1.8 × 10^−13^)
- Visual distortions at 405 ms post-stimulus (rho: +0.63; *p*-value: 1.1 × 10^−^ (Lu et al., 2021))
- Visual naming error: no significant correlation
- Speech arrest: 380 ms pre-response onset (rho: +0.56; *p*-value: 1.0 × 10^−6^)
- Facial sensorimotor: 65 ms post-response onset (rho: +0.54; *p*-value: 2.0 × 10^−6^).

Afferent and efferent flow intensities demonstrated similar patterns of correlation with symptom probability (eFigure 17).

### 3.9. Neural correlates of within-individual variability in response times in visual naming

During a period between 200 and 500 ms following stimulus onset, stronger efferent or afferent accelerating flows in the basal posterior temporal regions, including the bilateral pITG and the right parahippocampal gyrus were associated with faster responses (minimum rho: −1.0; *p*-value: 0.003; Fig. 8A).

## 4. Discussion

### 4.1. Summary

This study provides evidence that transient neural information flow through white matter corresponds to distinct stages of speech processing, as demonstrated by (1) the temporal evolution and directionality of information flow; (2) the association between flow strength and stimulation-induced symptoms; and (3) the association between flow strength and subsequent response time. A key distinction between the present study and our prior report (Kochi et al., 2026) lies in the characterization of directionality. In our previous study (Kochi et al., 2026), we identified time windows during which pairs of cortical regions connected by direct white matter pathways exhibited simultaneous high-gamma augmentation, thereby defining the spatiotemporal architecture of functional coactivation. In contrast, the current study employed transfer entropy–based effective connectivity analysis to determine the direction and timing of neural information flow between these regions. Accelerating information flow propagated from sensory to higher-order regions, became bidirectional, and was followed by decelerating phases. Neural information flow at specific time points predicted stimulation-induced speech symptoms. Specifically, information flow around visual naming response onset was significantly associated with stimulation-induced speech arrest and face sensorimotor symptoms, whereas coactivation measures were not (Kochi et al., 2026). Stronger information flow was also associated with faster responses within left perisylvian regions during auditory naming and within bilateral posterior basal temporal cortices during visual naming. Together, these findings indicate that directional neural information flow through white matter provides complementary insights into the sequential recruitment of large-scale brain networks underlying speech processing.

### 4.2. Auditory naming

Our findings revealed a spatiotemporal sequence of accelerating flows consistent with the dual-stream model of speech processing (Hickok and Poeppel, 2007; Dick and Tremblay, 2012; Chang et al., 2015), which proposes reciprocal interactions among auditory, temporal, and frontal cortices. At 45 ms after stimulus onset, unidirectional accelerating flow emerged from the left pSTG to the pMTG via U-fibers (Fig. 2A). By 55 ms, additional flow from the pSTG to the inferior precentral gyrus arose through the arcuate fasciculus (Fig. 2B). Regions generating stronger flows were associated with a higher probability of stimulation-induced auditory hallucinations (Fig. 3A). These early pSTG-originating flows likely reflect feedforward transmission to two parallel pathways: a ventral temporal stream supporting phonetic-to-semantic transformation (Conroy et al., 2018; Forseth et al., 2018; Binder et al., 2025) and a dorsal frontal stream mediating auditory–motor mapping via the arcuate fasciculus (Cogan et al., 2014). Around 200 ms, reverse-direction accelerating flows toward the pSTG emerged, consistent with feedback from perisylvian regions refining auditory representations (Holdgraf et al., 2016; Ozker et al., 2024). Given their early timing, these recurrent interactions may operate at a sublexical phonological level to optimize speech perception.

Variations in neural flow during auditory stimulus presentation may contribute to within-individual response time differences. Increased afferent decelerating flow to the right pSTG around 400 ms was associated with slower naming, whereas faster responses corresponded to reduced decelerating inflow (Fig. 4A). Consistent with this, our prior study showed that faster responses were accompanied by greater high-gamma augmentation in the right pSTG during the same interval (Kochi et al., 2026). These findings suggest that delayed responses may reflect reduced attention to auditory input, resulting in diminished neural gain and attenuated processing (Nourski et al., 2017).

Acceleration and sustained engagement of left perisylvian interactions facilitated transformation of semantic representations into articulatory plans. Stronger accelerating flows between the left pMTG and inferior precentral gyrus 250 ms before stimulus offset were associated with faster auditory naming (Fig. 4B), marking a convergence phase between ventral semantic regions and dorsal frontal areas supporting lexical retrieval and motor preparation. Regions exhibiting stronger accelerating flows were more likely to produce stimulation-induced receptive and expressive aphasia (Figs. 3B-3C), indicating their necessity for lexical access and fluent production. Consistently, our prior study showed that stronger coactivation across the left perisylvian network around stimulus offset predicted faster responses (Kochi et al., 2026). These regions are interconnected via the arcuate fasciculus, which supports bidirectional exchange between semantic-lexical and motor systems (Sonoda et al., 2021; Catani and Mesulam, 2008; Matsumoto et al., 2004); damage to this tract causes naming deficits (Gajardo-Vidal et al., 2021). These findings support posterior temporal–inferior frontal coordination via a dorsal stream pathway during auditory naming, addressing a question raised by Dick and Tremblay (Dick and Tremblay, 2012).

Our findings support Binder’s model proposing that the left pMTG serves as a semantic hub integrating phonological and syntactic inputs through reciprocal exchanges within perisylvian cortex (Binder, 2017). We extend this model by specifying the timing of accelerated bidirectional interactions involving the pMTG (Fig. 2) and demonstrating that its coupling with frontal perisylvian regions is reciprocal—primarily via the arcuate fasciculus—rather than strictly temporal-to-frontal. This bidirectional architecture likely reflects ongoing feedback between semantic integration and response selection that enables real-time updating of lexical meaning during speech comprehension (Murphy et al., 2023; Schroën et al., 2023).

Decelerating flows following accelerating phases may reflect a coordinated mechanism that redistributes neural resources across stages of speech processing. Before stimulus offset, accelerating flows along the left arcuate fasciculus were associated with stimulation-induced receptive and expressive aphasia (Figs. 3B-3C), implicating this tract in lexical-semantic retrieval. After offset, decelerating flows emerged along the same pathway, while accelerating flows arose via U-fibers linking left frontal and Rolandic regions, corresponding to stimulation-induced speech arrest (Fig. 3D). This acceleration–deceleration sequence may represent transient gating that attenuates semantic processing to permit motor recruitment for speech initiation. Consistent with prior reports of reciprocal task-positive and default mode activation (Kucewicz et al., 2024; Pinheiro-Chagas et al., 2024), our findings demonstrate temporally ordered opposing flows along the same eloquent pathways. This pattern aligns with the multiple-network model (Hagoort, 2019; Woolnough et al., 2023), in which speech production arises from coordinated transitions across distributed systems spanning conceptual access to articulation.

### 4.3. Visual naming

Visual naming engaged both ventral and dorsal streams, with ventral activation preceding dorsal involvement. At 65 ms after picture onset, unidirectional accelerating flow emerged from the left lateral occipital gyrus to the posterior fusiform gyrus via the inferior longitudinal fasciculus (Fig. 6A). Stronger early efferent flows were associated with stimulation-induced phosphenes and visual distortions (Figs. 7A-7B), consistent with feedforward transmission from lower-order visual regions to ventral temporal areas supporting hierarchical object processing (Forseth et al., 2018; Tang et al., 2014). Around 400 ms, bidirectional accelerating flows appeared along both streams, marking the transition from object recognition to lexical–semantic processing and early articulatory planning. In the ventral stream, flows propagated bilaterally through the inferior longitudinal fasciculus between occipital and basal temporal regions (Figs. 6A-6B), with stronger efferent flows from these areas predicting faster naming (Fig. 8A). In the dorsal stream, bidirectional flows emerged through the left superior longitudinal fasciculus linking lateral occipital and frontal regions (Fig. 5B), and via the arcuate fasciculus between the left pITG and inferior precentral gyrus (Fig. 6C), bypassing the pSTG and pMTG. These findings indicate that visual and auditory naming rely on distinct dorsal stream configurations, addressing a question raised previously (Dick and Tremblay, 2012).

Frontal engagement likely reflects motor preparation integrating lexical retrieval with articulatory planning. Regions exhibiting stronger accelerating flows around 400 ms post-stimulus were associated with stimulation-induced visual distortions (Fig. 7B), and stimulation of the left fusiform gyrus and pITG frequently produced picture-naming errors (eFigure 15) (Forseth et al., 2018). This timing aligns with N400-related semantic processing observed in healthy individuals (Aristei et al., 2011).

During the first 300 ms after picture onset, we found no evidence of top-down modulation from Broca’s area. Bidirectional decelerating flows appeared across the left IFG, rostral middle frontal gyrus, and medial occipital gyri (Fig. 5A), suggesting transient frontal suppression. Neural processing at this stage was dominated by feedforward activity within posterior object-recognition networks. Consistently, visual naming response times were unrelated to flows involving the left IFG, whereas faster auditory naming was associated with greater left IFG coactivation near stimulus offset (Kochi et al., 2026). These findings indicate that visual naming initially relies on posterior ventral-stream mechanisms, with frontal regions supporting lexical retrieval (Schnur et al., 2009) and motor initiation (Tate et al., 2014; Nakai et al., 2017; Lu et al., 2021) engaged only at later stages.

Before responses, accelerating flows shifted to frontal regions initiating articulation. Bidirectional interactions emerged among the left IFG, pSFG, and inferior precentral gyrus via U-fibers and the frontal aslant fasciculus (Figs. 6D-6E). Concurrent coactivation between the left IFG and pITG through the arcuate fasciculus (Fig. 5C) linked lexical retrieval with motor planning. Accelerating flows during this phase were associated with stimulation-induced speech arrest (Fig. 7D), consistent with a transition from lexical to articulatory processing (Flinker et al., 2015; Schnur et al., 2009).

### 4.4. Utility

eVideos 1-4 illustrate two temporally resolved interaction types: directional flow and coactivation. Although validation with postoperative neuropsychological outcomes is pending, both maps demonstrated functional relevance. During auditory naming, accelerating flow and coactivation similarly predicted stimulation-induced symptoms (Spearman’s rho ≈ 0.6–0.8) (Kochi et al., 2026). In visual naming, both measures tracked visual phenomena such as phosphenes and distortions (rho ≈ 0.6–0.9).

However, a mechanistic dissociation emerged. During visual naming, only accelerating flows—and not coactivation intensity—predicted speech arrest and face sensorimotor symptoms (Figs. 7D-7E). Persistent occipital coactivation near response onset may obscure such associations, whereas transient accelerating flows more precisely index motor initiation. Surges in both flow and coactivation measures were associated with faster naming, though fewer regions correlated with accelerating flow, consistent with its phasic nature compared with sustained coactivation (Kochi et al., 2026). Our results highlight the complementary value of the two maps: accelerating flow maps capture rapid, stage-specific signaling, whereas coactivation maps reflect broader network integration during speech processing.

### 4.5. Limitations

In the present study, we did not directly measure neural signal propagation through specific white matter tracts. Rather, we inferred that neural information propagated through white matter directly connecting two cortical regions when transfer entropy–based effective connectivity analysis indicated that high-gamma activity in one cortical region predicted subsequent high-gamma activity in another. We did not model non–white matter-mediated cortico-cortical interactions, such as local intracortical propagation through the cortical gray matter, which may involve horizontal cortical fibers, recurrent local circuitry, and polysynaptic transmission in addition to direct long-range axonal transmission (Roland et al., 2014). Future studies using high-density intracranial electrode arrays are warranted to determine the relative contributions of white matter-mediated and non–white matter-mediated interactions to cortical information transfer.

We did not analyze neural information flows within individual patients. Although within-patient iEEG analyses are well suited to determining the temporal sequence of local high-gamma modulation between electrode sites, comprehensive bilateral sampling of nonepileptic cortex is neither ethical nor practical. Consequently, individual datasets cannot provide complete coverage of cortical systems. Instead, our group-level approach was designed to model neural interactions at the population level rather than within individual patients. Key ROIs were sampled in >20 patients and included >70 electrodes each (eTable 2). Leave-one-out analyses demonstrated that no single patient disproportionately influenced the results (Spearman's rho, 0.95–0.99), and the narrow confidence intervals further supported the robustness and generalizability of the findings (eFigures 3-4).

This study is limited by the spatial constraints of intracranial EEG, which preclude sampling of subcortical structures such as the thalamus. Accordingly, we could not assess cortico-thalamic or thalamo-cortical neural interactions. Furthermore, we considered transfer entropy only between cortical ROI pairs directly connected by white matter streamlines. The present study was not designed to model indirect neural interactions mediated through multiple white matter pathways. Given that our atlas included 66 ROIs (Kochi et al., 2026), there were up to 274,560 possible ordered ROI triplets. Systematically evaluating and visualizing indirect neural information flow across such higher-order pathways would be computationally and analytically prohibitive and was therefore beyond the scope of the present study.

Indirect propagation through intermediate regions is a potential source of spurious connectivity in network analyses. We sought to minimize this risk in two ways. First, neural information flow was defined only between cortical regions connected by a direct white matter pathway. Thus, even if high-gamma activity at ROI_1_ predicted subsequent activity at ROI_2_, information flow was not declared unless a direct white matter streamline connected the two regions in the structural connectivity atlas. Second, information flow was identified only when transfer entropy detected high-gamma acceleration at ROI_1_ preceding that at ROI_2_ across delay parameters of 5–15 ms. Prior single-pulse electrical stimulation studies indicate that direct white matter–mediated propagation between distinct cortical gyri typically occurs within approximately 10–50 ms (Matsumoto et al., 2004), whereas indirect propagation through intermediate regions is expected to require longer delays. Accordingly, restricting the analysis to short delay parameters was intended to preferentially identify direct rather than indirect neural interactions.

Although analyses were restricted to non-epileptic sites (Sakakura et al., 2023), we examined whether remote effects of focal epilepsy influenced our findings. Patients with left-hemisphere seizure foci exhibited reduced leftward lateralization of neural interactions (eResults), consistent with previous reports (Kochi et al., 2026). Nonetheless, these effects did not alter our principal conclusions regarding the large-scale spatiotemporal organization of neural information flow. Furthermore, age was not independently associated with local high-gamma amplitude after adjustment for seizure focus laterality and handedness. We cannot exclude the possibility that more subtle developmental effects remained undetected.

Some readers may wonder why neural information flow is transient, whereas functional coactivation is more sustained (eVideos 1 and 2). This difference may reflect the temporal characteristics of the underlying high-gamma measures. Neural information flow was defined by high-gamma acceleration, which was typically transient, whereas functional coactivation was defined by significant high-gamma augmentation relative to the prestimulus baseline, which often persisted substantially longer. For example, high-gamma augmentation in the left lateral occipital gyrus lasted more than one second, whereas high-gamma acceleration in the same region lasted <400 ms (eFigure 4). Consequently, neural information flow was more temporally restricted than functional coactivation.

We caution against interpretation of interactions distant from stimulus or response timing, as inter-individual variability may reduce temporal precision. However, confidence intervals did not widen substantially in these windows (eFigures 3-4), suggesting stable group-level estimates. Electrical stimulation mapping validated the functional relevance of our maps.

## Supplementary Material

1

2

3

4

5

6

7

8

Supplementary material associated with this article can be found, in the online version, at doi:10.1016/j.neuroimage.2026.122236.

## Figures and Tables

**Fig. 1.**
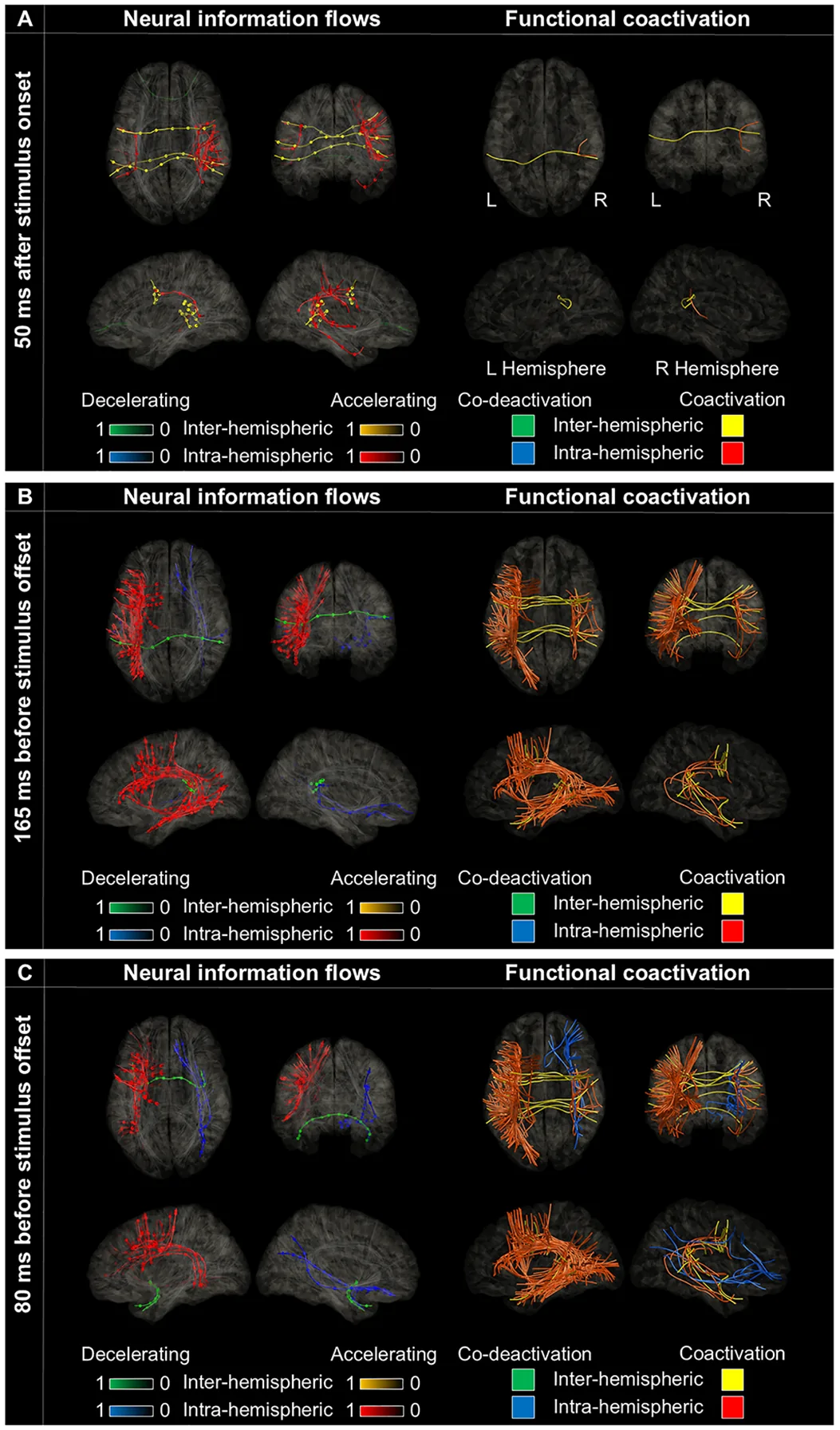

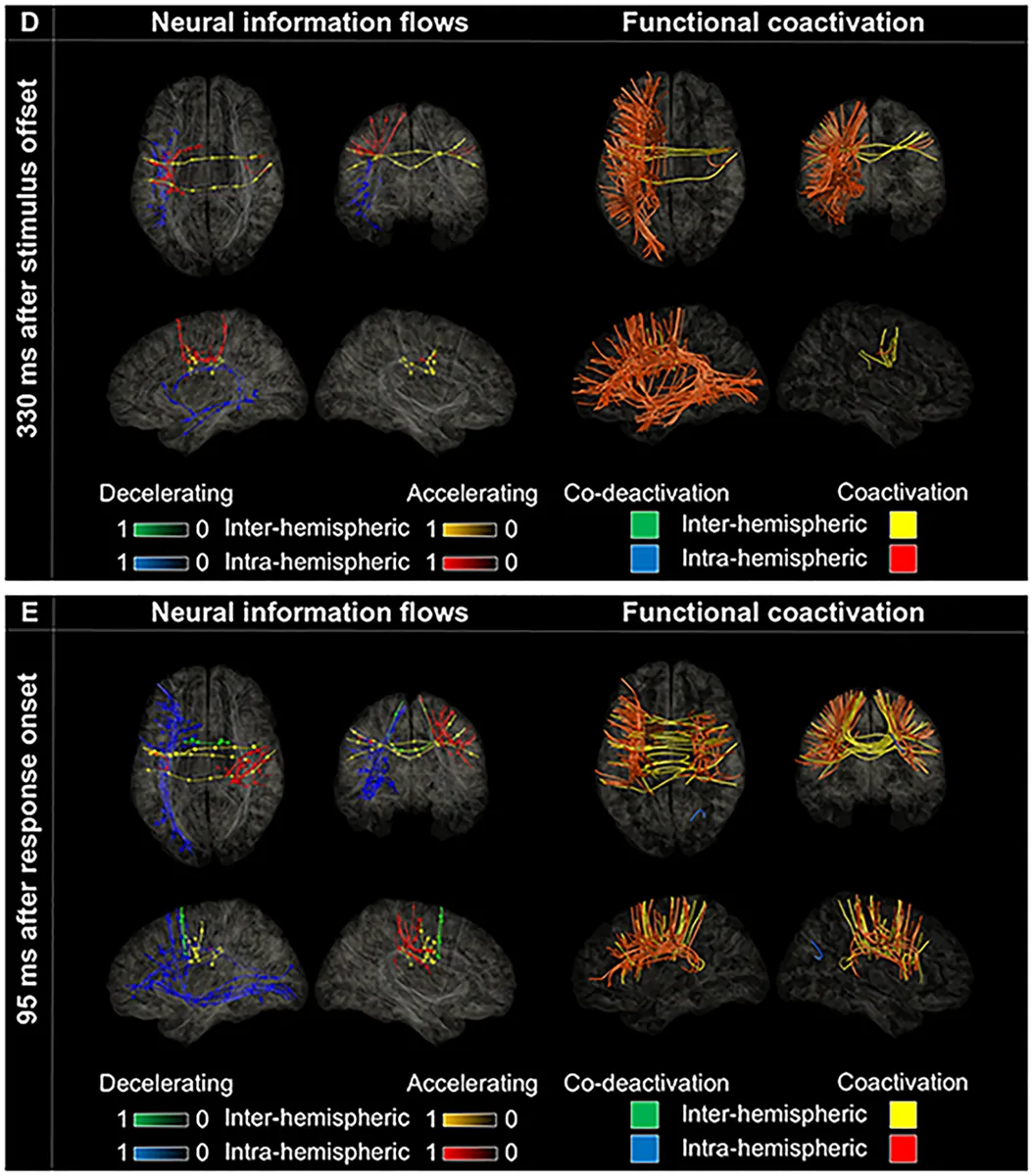
Neural information flows and functional coactivation/co-deactivation during auditory naming. Left: Accelerating (red/yellow) and decelerating (blue/green) flows through white matter tracts. Right: Functional coactivation (red/yellow) and co-deactivation (blue/green). **A** 50 ms after stimulus onset, efferent accelerating flow intensity at a given ROI correlated with probability of stimulation-induced auditory hallucinations. **B** 165 ms before stimulus offset, both efferent and afferent flows correlated with receptive aphasia. **C** 80 ms before stimulus offset, both efferent and afferent flows correlated with expressive aphasia. **D** 330 ms after stimulus offset, both efferent and afferent flows correlated with speech arrest. **E** 95 ms after response onset, both efferent and afferent flows correlated with face sensorimotor symptoms. L: left. R: right.

**Fig. 2.**
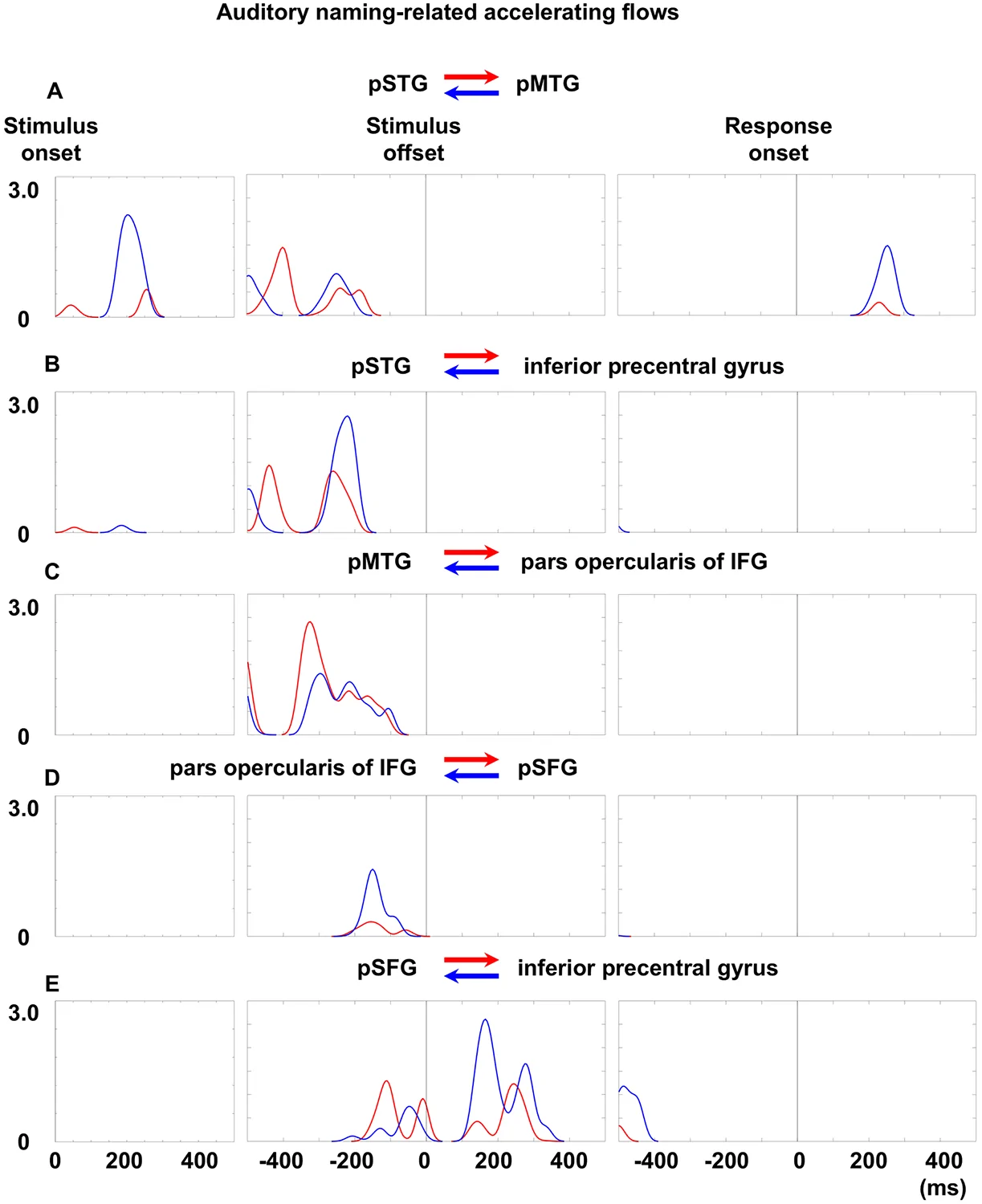
Auditory naming–related accelerating flows within the left hemisphere. This figure demonstrates that the accelerating flows originating from the perceptual region were initially unidirectional, whereas subsequent flows were predominantly bidirectional. *X*-axis: time; *Y*-axis: flow intensity (transfer entropy). Plot colors denote the direction of accelerating flows between the following regions of interest. **A** posterior superior temporal gyrus (pSTG) and posterior middle temporal gyrus (pMTG). **B** pSTG and inferior precentral gyrus. **C** pMTG and pars opercularis of the inferior frontal gyrus (IFG). **D** pars opercularis of the IFG and posterior superior frontal gyrus (pSFG). **E** pSFG and inferior precentral gyrus. eVideos 1 and 3 summarize neural information flow at the whole-brain level.

**Fig. 3.**
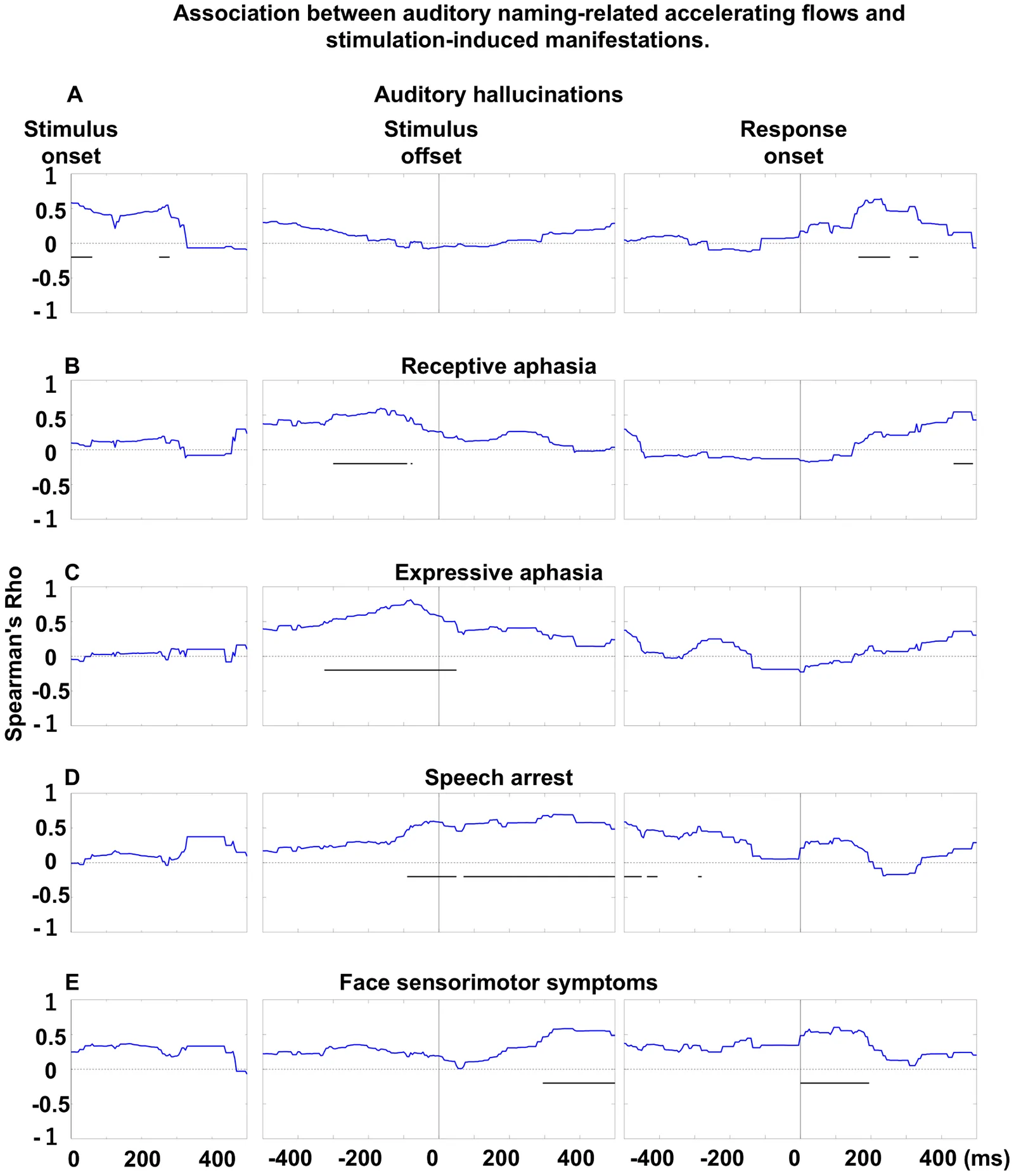
Association between auditory naming–related accelerating flows and stimulation-induced manifestations. Each plot shows Spearman’s rho, reflecting the correlation between mean efferent flow intensity and the likelihood of stimulation-induced symptoms across time bins. **A-E** Correlations between flows and stimulation-induced manifestations. Rho values > 0 indicate that, at a given moment, the probability of a specific manifestation was higher in regions emitting accelerating flows to other regions via white matter tracts, as determined by transfer entropy–based effective connectivity analysis. Horizontal bars mark intervals of significant correlation. Spearman’s rho was not computed for time bins lacking neural information flows. For example, Fig. 3B and C demonstrate that regions emitting stronger accelerating flows before auditory stimulus offset were associated with a higher probability of eliciting receptive or expressive aphasia during electrical stimulation.

**Fig. 4.**
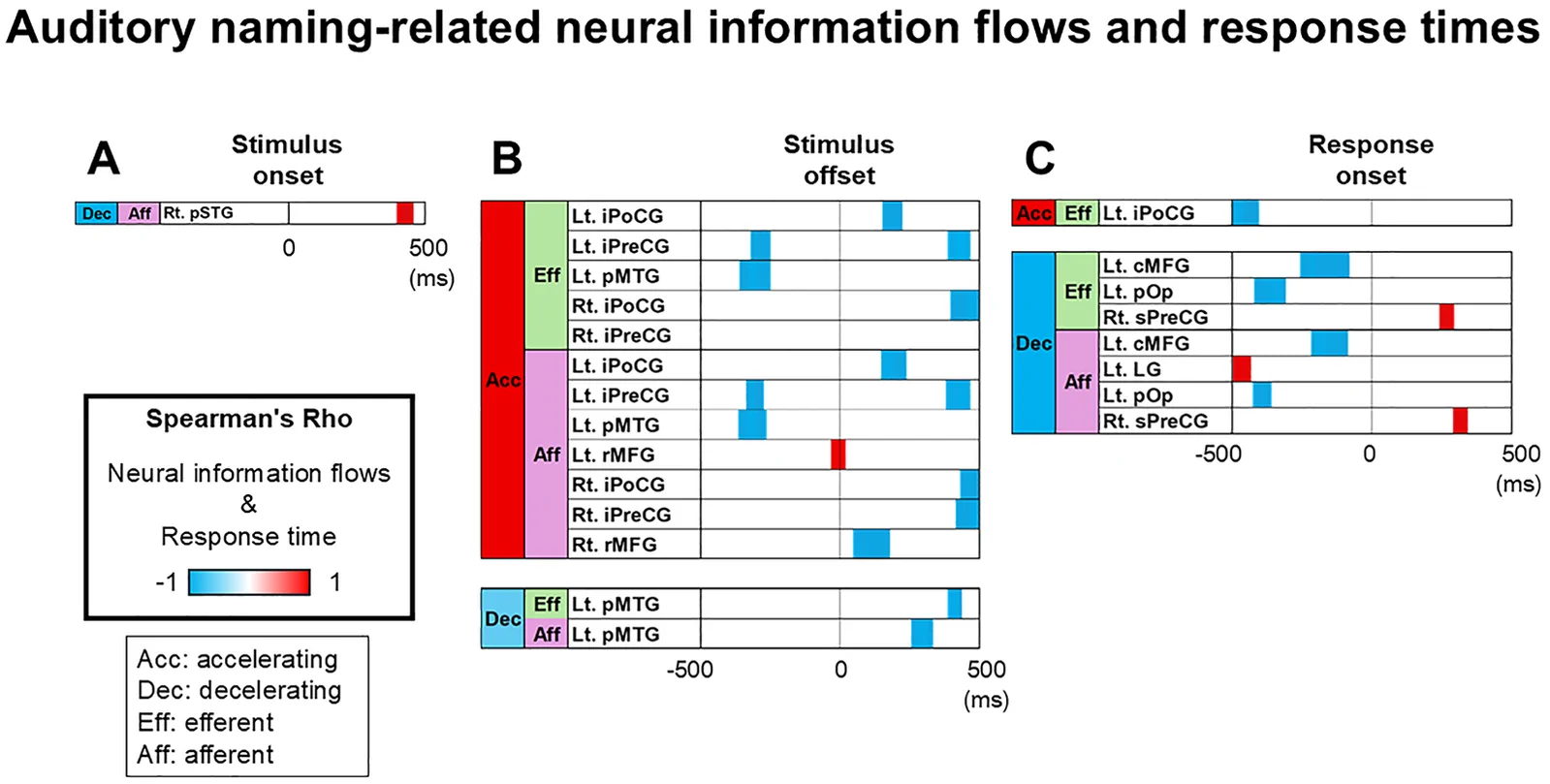
Auditory naming-related neural information flows and response times. Spearman’s rho over time illustrating correlations between neural information flow intensity and naming response times. Blue indicates increased flows associated with faster responses; red indicates slower responses. Only regions of interest (ROIs) with significant correlations are shown. Significance was defined as *p*-value < 0.05 sustained across a 50-ms period. **A** following stimulus onset. **B** around stimulus offset. **C** around response onset. Full names of all regions of interest are provided in eTable 2. For example, Fig. 4B demonstrates that trials with stronger efferent and afferent flows in the left inferior precentral gyrus (iPreCG) and posterior middle temporal gyrus (pMTG) before auditory stimulus offset were associated with faster naming responses.

**Fig. 5.**
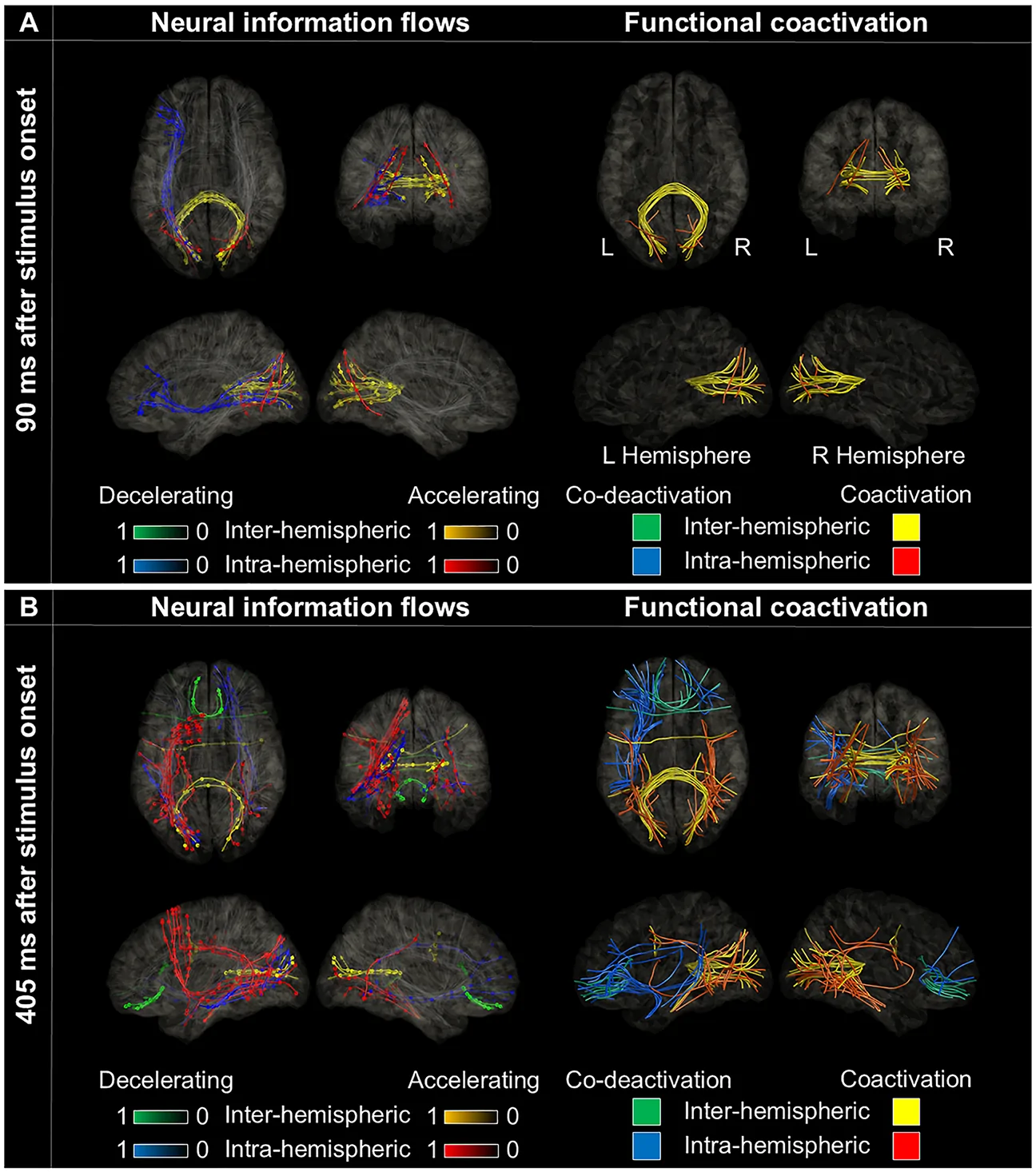

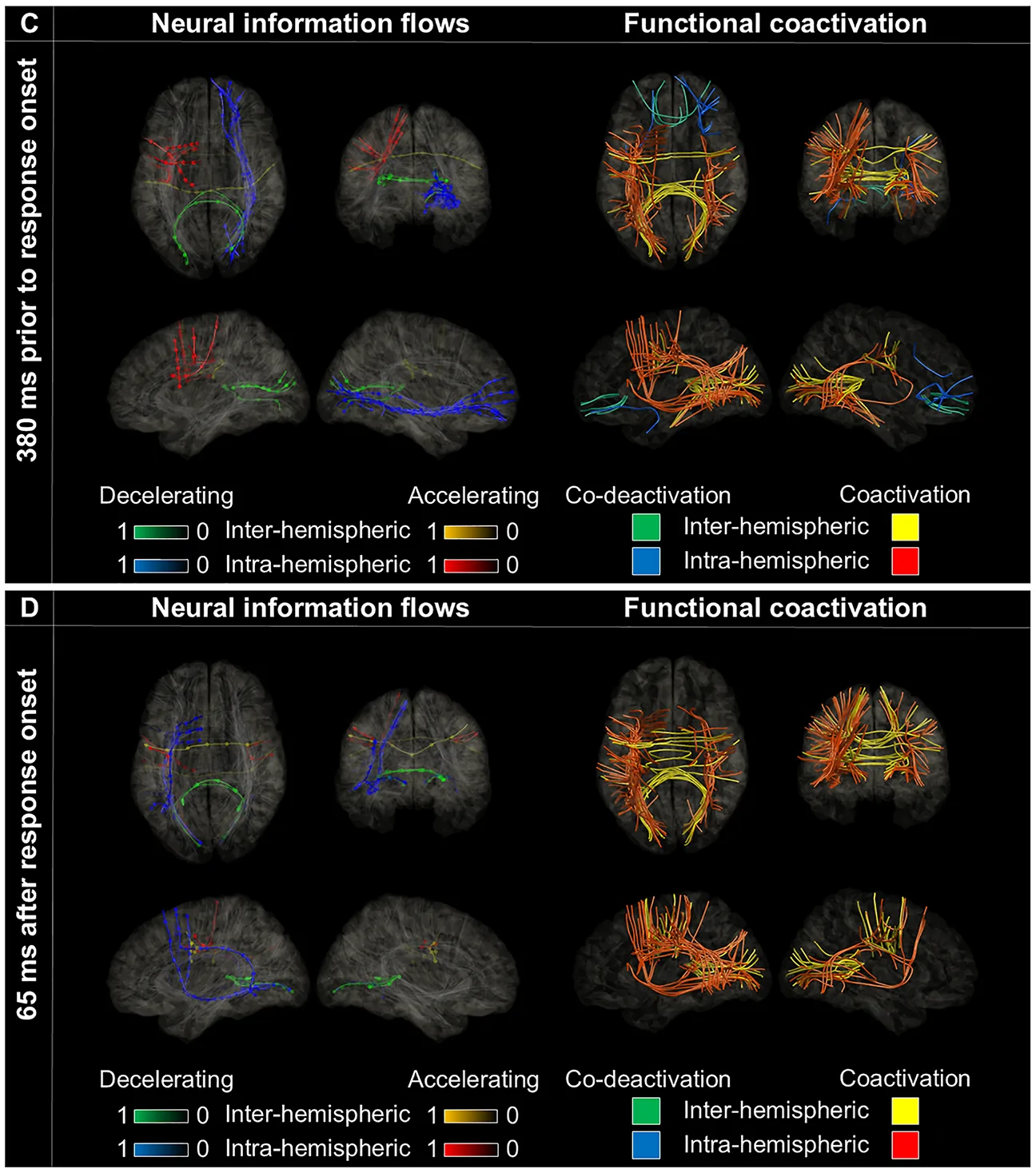
Neural information flows and functional coactivation/co-deactivation during visual naming. **A** 90 ms after stimulus onset, both efferent and afferent accelerating flow intensity correlated with stimulation-induced phosphene and visual distortion. **B** 405 ms after stimulus onset, both efferent and afferent flow intensity correlated with stimulation-induced visual distortion. **C** 380 ms prior to response onset, both efferent and afferent flow intensity correlated with speech arrest. **D** 65 ms after response onset, both efferent and afferent flow intensity correlated with face sensorimotor symptoms. L: left. R: right.

**Fig. 6.**
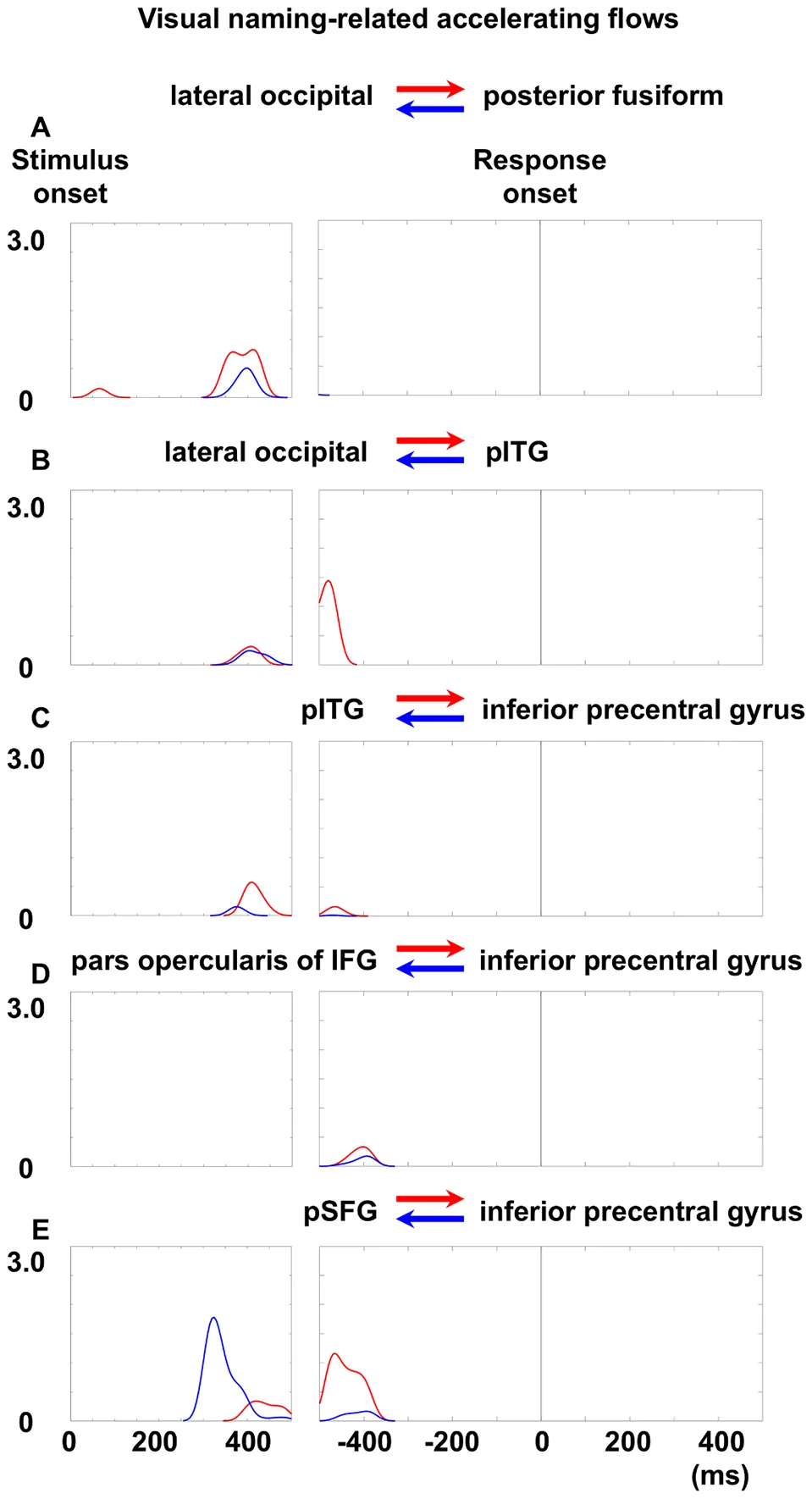
Visual naming–related accelerating flows within the left hemisphere. This figure demonstrates that the accelerating flows from the lateral occipital region to the posterior fusiform region were initially unidirectional, whereas subsequent flows were predominantly bidirectional. **A** lateral occipital and posterior fusiform gyri. **B** lateral occipital and posterior inferior temporal gyrus (pITG). **C** pITG and inferior precentral gyrus. **D** pars opercularis of the IFG and inferior precentral gyrus. **E** posterior superior frontal gyrus (pSFG) and inferior precentral gyrus. eVideos 2 and 4 summarize neural information flow at the whole-brain level.

**Fig. 7.**
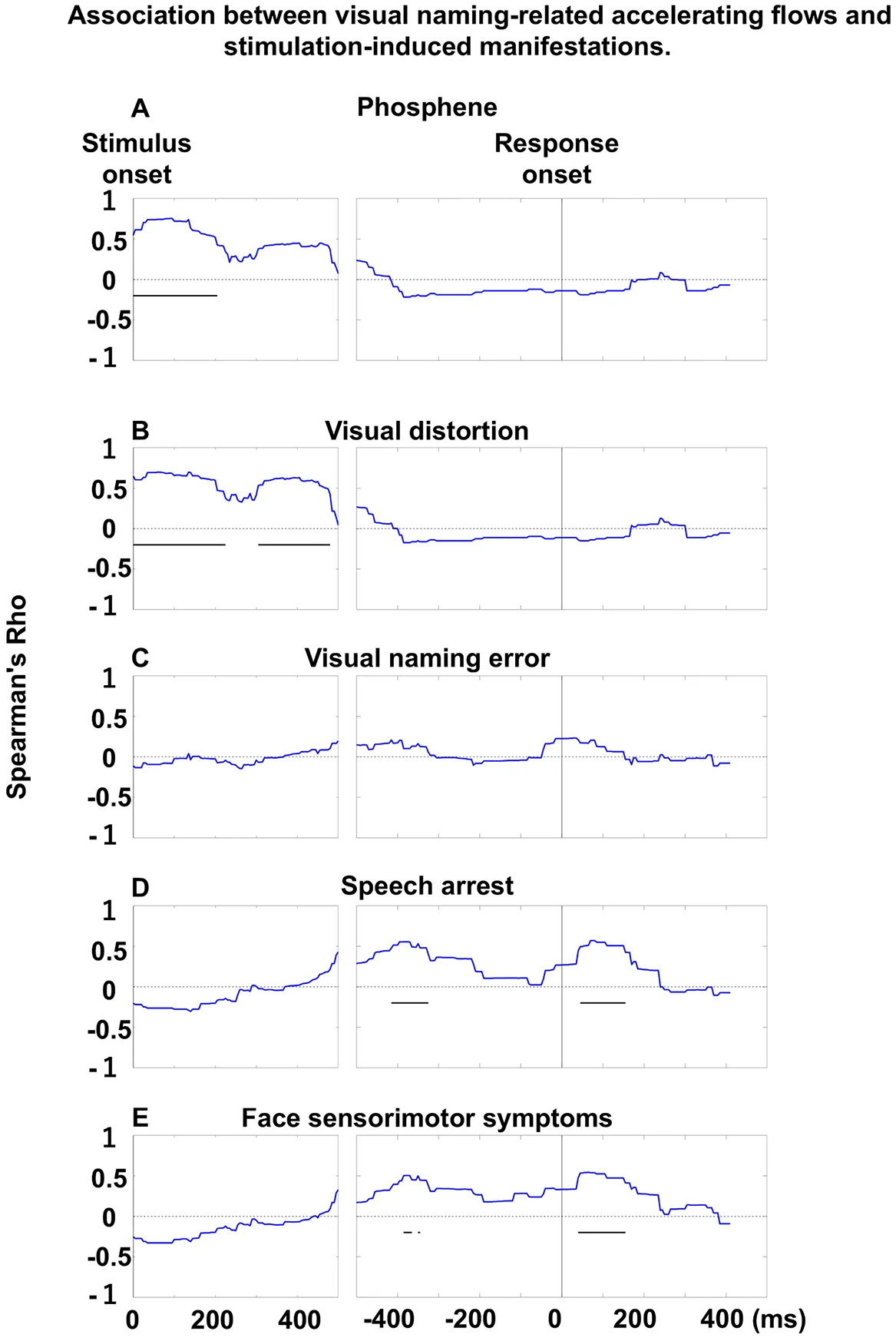
Association between visual naming–related accelerating flows and stimulation-induced manifestations. Each plot shows Spearman’s rho, reflecting the correlation between mean flow intensity and the likelihood of stimulation-induced symptoms across time bins. **A–E** Correlations between efferent accelerating flows and stimulation-induced manifestations. Rho values > 0 indicate that, at a given moment, the probability of a given manifestation was higher in regions emitting flows to other regions via white matter tracts, as determined by transfer entropy–based effective connectivity analysis. Horizontal bars mark intervals of significant correlation. Spearman’s rho was not computed for time bins lacking neural information flows. For example, Fig. 7D demonstrates that regions emitting stronger accelerating flows before and after response onset were associated with a higher probability of eliciting speech arrest during electrical stimulation.

**Fig. 8.**
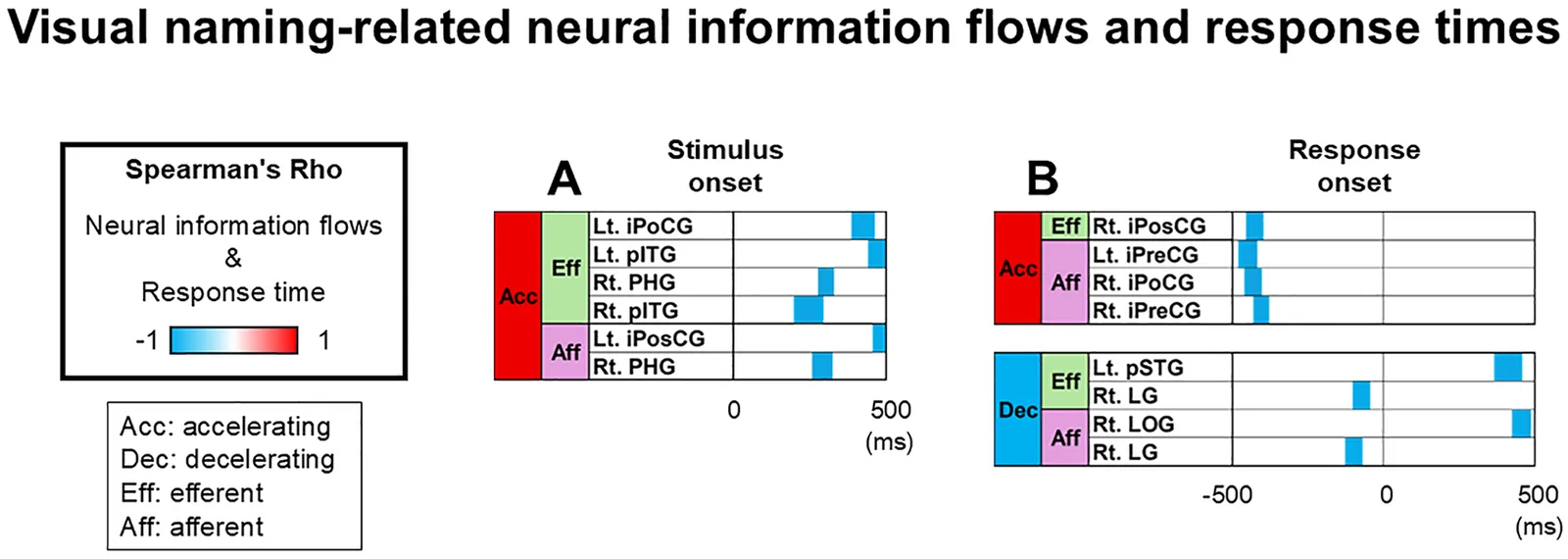
Visual naming-related neural information flows and response times. Spearman’s rho over time illustrating correlations between neural information flow intensity and naming response times. Blue indicates increased flows associated with faster responses; red indicates slower responses. **A** following stimulus onset. **B** around response onset. Full names of all regions of interest are provided in eTable 2. For example, Fig. 8A demonstrates that trials with stronger efferent and afferent flows in the right parahippocampal gyrus (PHG), as well as stronger efferent flows in the right or left posterior inferior temporal gyrus (pITG), after visual stimulus onset were associated with faster naming responses.

## References

[R1] Aristei S, Melinger A, Abdel Rahman R, 2011. Electrophysiological chronometry of semantic context effects in language production. J. Cogn. Neurosci. 23, 1567–1586. 10.1162/jocn.2010.21474 20515409

[R2] Binder JR, 2017. Current controversies on Wernicke's area and its role in language. Curr. Neurol. Neurosci. Rep. 17, 58. 10.1007/s11910-017-0764-8 28656532

[R3] Binder JR, Freiberg A, Heffernan J, , 2025. Lesion correlates of impaired acoustic-phonetic perception after unilateral left hemisphere stroke. Brain 148, 1421–1434. 10.1093/brain/awae417 PMC12168130 39789712

[R4] Burke JF, Long NM, Zaghloul KA, , 2014. Human intracranial high-frequency activity maps episodic memory formation in space and time. Neuroimage 85, 834–843. 10.1016/j.neuroimage.2013.06.067 PMC4289670 23827329

[R5] Catani M, Mesulam M, 2008. The arcuate fasciculus and the disconnection theme in language and aphasia: history and current state. Cortex 44, 953–961. 10.1016/j.cortex.2008.04.002 PMC2740371 18614162

[R6] Chang EF, Raygor KP, Berger MS, 2015. Contemporary model of language organization: an overview for neurosurgeons. J. Neurosurg. 122, 250–261. 10.3171/2014.10.JNS132647 25423277

[R7] Cogan GB, Thesen T, Carlson C, , 2014. Sensory-motor transformations for speech occur bilaterally. Nature 507, 94–98. 10.1038/nature12935 PMC4000028 24429520

[R8] Conroy P, Sotiropoulou Drosopoulou C, Humphreys GF, , 2018. Time for a quick word? The striking benefits of training speed and accuracy of word retrieval in post-stroke aphasia. Brain 141, 1815–1827. 10.1093/brain/awy087 29672757

[R9] Desikan RS, Ségonne F, Fischl B, , 2006. An automated labeling system for subdividing the human cerebral cortex on MRI scans into gyral based regions of interest. Neuroimage 31, 968–980. 10.1016/j.neuroimage.2006.01.021 16530430

[R10] Dick AS, Tremblay P, 2012. Beyond the arcuate fasciculus: consensus and controversy in the connectional anatomy of language. Brain 135, 3529–3550. 10.1093/brain/aws222 23107648

[R11] Ervin B, Frink C, Buroker J, , 2025. Behavior-iEEG-spectral-power-correlation: defining neural substrates of naturalistic behavior. Ann. Neurol. 98, 1096–1110. 10.1002/ana.70002 40817733

[R12] Firestone E, Uda H, Kuroda N, , 2025. Normative high-frequency oscillation phase-amplitude coupling and effective connectivity under sevoflurane. Brain Commun. 7, fcaf403. 10.1093/braincomms/fcaf403 PMC12582361 41189957

[R13] Flinker A, Korzeniewska A, Shestyuk AY, , 2015. Redefining the role of Broca's area in speech. Proc. Natl. Acad. Sci. USA 112, 2871–2875. 10.1073/pnas.1414491112 PMC4352780 25730850

[R14] Forseth KJ, Kadipasaoglu CM, Conner CR, , 2018. A lexical semantic hub for heteromodal naming in middle fusiform gyrus. Brain 141, 2112–2126. 10.1093/brain/awy120 PMC6365955 29860298

[R15] Gajardo-Vidal A, Lorca-Puls DL, Team P, , 2021. Damage to Broca's area does not contribute to long-term speech production outcome after stroke. Brain 144, 817–832. 10.1093/brain/awaa460 PMC8041045 33517378

[R16] Hagoort P, 2019. The neurobiology of language beyond single-word processing. Science 366, 55–58. 10.1126/science.aax0289 31604301

[R17] Hickok G, Poeppel D, 2007. The cortical organization of speech processing. Nat. Rev. Neurosci. 8, 393–402. 10.1038/nrn2113 17431404

[R18] Hoechstetter K, Bornfleth H, Weckesser D, , 2004. BESA source coherence: a new method to study cortical oscillatory coupling. Brain Topogr. 16, 233–238. 10.1023/b:brat.0000032857.55223.5d 15379219

[R19] Holdgraf CR, de Heer W, Pasley B, , 2016. Rapid tuning shifts in human auditory cortex enhance speech intelligibility. Nat. Commun. 7, 13654. 10.1038/ncomms13654 PMC5187445 27996965

[R20] Ito S, Hansen ME, Heiland R, , 2011. Extending transfer entropy improves identification of effective connectivity in a spiking cortical network model. PLoS ONE 6, e27431. 10.1371/journal.pone.0027431 PMC3216957 22102894

[R21] Kanno A, Kochi R, Sakakura K, , 2025. Dynamic causal tractography analysis of auditory descriptive naming: an intracranial study of 106 patients. Neuroimage 317, 121319. 10.1016/j.neuroimage.2025.121319 PMC12355976 40516665

[R22] Kochi R, Kanno A, Uda H, , 2026. Whole-brain dynamic causal tractography atlas of auditory and visual speech networks derived from intracranial neurophysiological data. Neurology 106, e2144. 10.1212/WNL.0000000000214448 PMC12746732 41453123

[R23] Kucewicz MT, Cimbalnik J, Garcia-Salinas JS, , 2024. High frequency oscillations in human memory and cognition: a neurophysiological substrate of engrams? Brain 147, 2966–2982. 10.1093/brain/awae159 PMC11370809 38743818

[R24] Lu J, Zhao Z, Zhang J, , 2021. Functional maps of direct electrical stimulation-induced speech arrest and anomia: a multicentre retrospective study. Brain 144, 2541–2553. 10.1093/brain/awab125 PMC8453410 33792674

[R25] Matsumoto R, Nair DR, LaPresto E, , 2004. Functional connectivity in the human language system: a cortico-cortical evoked potential study. Brain 127, 2316–2330. 10.1093/brain/awh246 15269116

[R26] Möddel G, Lineweaver T, Schuele SU, , 2009. Atypical language lateralization in epilepsy patients. Epilepsia 50, 1505–1516. 10.1111/j.1528-1167.2008.02000.x 19243420

[R27] Murphy E, Forseth KJ, Donos C, , 2023. The spatiotemporal dynamics of semantic integration in the human brain. Nat. Commun. 14, 6336. 10.1038/s41467-023-42087-8 PMC10598228 37875526

[R28] Nakai Y, Jeong JW, Brown EC, , 2017. Three- and four-dimensional mapping of speech and language in patients with epilepsy. Brain 140, 1351–1370. 10.1093/brain/awx051 PMC5405238 28334963

[R29] Nir Y, Fisch L, Mukamel R, , 2007. Coupling between neuronal firing rate, gamma LFP, and BOLD fMRI is related to interneuronal correlations. Curr. Biol. 17, 1275–1285. 10.1016/j.cub.2007.06.066 17686438

[R30] Nishida M, Juhász C, Sood S. , 2008. Cortical glucose metabolism positively correlates with gamma-oscillations in nonlesional focal epilepsy. Neuroimage 42, 1275–1284. 10.1016/j.neuroimage.2008.06.027 PMC2614873 18652902

[R31] Nourski KV, Steinschneider M, Rhone AE, Howard III MA, 2017. Intracranial electrophysiology of auditory selective attention associated with speech classification tasks. Front. Hum. Neurosci. 10, 691. 10.3389/fnhum.2016.00691 PMC5222875 28119593

[R32] Ozker M, Yu L, Dugan P, , 2024. Speech-induced suppression and vocal feedback sensitivity in human cortex. Elife 13, RP94198. 10.7554/eLife.94198 PMC11386952 39255194

[R33] Perrone-Bertolotti M, Alexandre S, Jobb AS, , 2020. Probabilistic mapping of language networks from high frequency activity induced by direct electrical stimulation. Hum. Brain Mapp. 41, 4113–4126. 10.1002/hbm.25112 PMC7469846 32697353

[R34] Pinheiro-Chagas P, Sava-Segal C, Akkol S, , 2024. Spatiotemporal dynamics of successive activations across the human brain during simple arithmetic processing. J. Neurosci. 44, e2118222024. 10.1523/JNEUROSCI.2118-22.2024 PMC11044197 38485257

[R35] Ray S, Crone NE, Niebur E, , 2008. Neural correlates of high-gamma oscillations (60-200 Hz) in macaque local field potentials and their potential implications in electrocorticography. J. Neurosci. 28, 11526–11536. 10.1523/JNEUROSCI.2848-08.2008 PMC2715840 18987189

[R36] Roland PE, Hilgetag CC, Deco G, 2014. Cortico-cortical communication dynamics. Front. Syst. Neurosci. 8, 19. 10.3389/fnsys.2014.00019 PMC4017159 24847217

[R37] Sakakura K, Kuroda N, Sonoda M, , 2023. Developmental atlas of phase-amplitude coupling between physiologic high-frequency oscillations and slow waves. Nat. Commun. 14, 6435. 10.1038/s41467-023-42091-y PMC10575956 37833252

[R38] Schnur TT, Schwartz MF, Kimberg DY, , 2009. Localizing interference during naming: convergent neuroimaging and neuropsychological evidence for the function of Broca's area. Proc. Natl. Acad. Sci. USA 106, 322–327. 10.1073/pnas.0805874106 PMC2629229 19118194

[R39] Schreiber T, 2000. Measuring information transfer. Phys. Rev. Lett. 85, 461–464. 10.1103/PhysRevLett.85.461 10991308

[R40] Schroën JAM, Gunter TC, Numssen O, , 2023. Causal evidence for a coordinated temporal interplay within the language network. Proc. Natl. Acad. Sci. USA 120, e2306279120. 10.1073/pnas.2306279120 PMC10666120 37963247

[R41] Solomon EA, Kragel JE, Sperling MR, , 2017. Widespread theta synchrony and high-frequency desynchronization underlies enhanced cognition. Nat. Commun. 8, 1704. 10.1038/s41467-017-01763-2 PMC5700170 29167419

[R42] Sonoda M, Rothermel R, Carlson A, , 2022. Naming-related spectral responses predict neuropsychological outcome after epilepsy surgery. Brain 145, 517–530. 10.1093/brain/awab318 PMC9014727 35313351

[R43] Sonoda M, Silverstein BH, Jeong JW, , 2021. Six-dimensional dynamic tractography atlas of language connectivity in the developing brain. Brain 144, 3340–3354. 10.1093/brain/awab225 PMC8677551 34849596

[R44] Tang H, Buia C, Madhavan R, , 2014. Spatiotemporal dynamics underlying object completion in human ventral visual cortex. Neuron 83, 736–748. 10.1016/j.neuron.2014.06.017 PMC4134509 25043420

[R45] Tate MC, Herbet G, Moritz-Gasser S, , 2014. Probabilistic map of critical functional regions of the human cerebral cortex: Broca's area revisited. Brain 137, 2773–2782. 10.1093/brain/awu168 24970097

[R46] Thesen T, McDonald CR, Carlson C, , 2012. Sequential then interactive processing of letters and words in the left fusiform gyrus. Nat. Commun. 3, 1284. 10.1038/ncomms2220 PMC4407686 23250414

[R47] Woolnough O, Donos C, Murphy E, , 2023. Spatiotemporally distributed frontotemporal networks for sentence reading. Proc. Natl. Acad. Sci. USA 120, e2300252120. 10.1073/pnas.2300252120 PMC10151604 37068244

[R48] Woolnough O, Donos C, Rollo PS, , 2021. Spatiotemporal dynamics of orthographic and lexical processing in the ventral visual pathway. Nat. Hum. Behav. 5, 389–398. 10.1038/s41562-020-00982-w PMC10365894 33257877

[R49] Wurm MF, Caramazza A, 2022. Two 'what' pathways for action and object recognition. Trends. Cogn. Sci. 26, 103–116. 10.1016/j.tics.2021.10.003 34702661

[R50] Yeh FC, Panesar S, Fernandes D, , 2018. Population-averaged atlas of the macroscale human structural connectome and its network topology. Neuroimage 178, 57–68. 10.1016/j.neuroimage.2018.05.027 PMC6921501 29758339

