# Supplementary material for: Whole-brain millisecond-scale effective connectivity of auditory and visual naming
Source: Neuroimage. Author manuscript; Available in PMC 2026 Oct 13. (PMC13659335; doi:10.1016/j.neuroimage.2026.122236)
Supplement: Supplementary file 1 [file NIHMS2214451-supplement-1.docx]

**Supplementary Figures: eFigures**

**eFigure 1: Flowcharts showing the selection of patients who met eligibility criteria.**

**eFigure 2: Intracranial electrode coverage.**

**eFigure 3: Auditory naming-related high-gamma dynamics: means and 95% confidence intervals.**

**eFigure 4: Visual naming-related high-gamma dynamics: means and 95% confidence intervals.**

**eFigure 5: Auditory naming-related high-gamma dynamics: leave-one-patient-out analysis.**

**eFigure 6: Visual naming-related high-gamma dynamics: leave-one-patient-out analysis.**

**eFigure 7: Auditory naming-related accelerating flows through each fasciculus.**

**eFigure 8: Visual naming-related accelerating flows through each fasciculus.**

**eFigure 9: Auditory naming-related decelerating flows through each fasciculus.**

**eFigure 10: Visual naming-related decelerating flows through each fasciculus.**

**eFigure 11: Auditory naming-related functional coactivation through each fasciculus.**

**eFigure 12: Visual naming-related functional coactivation through each fasciculus.**

**eFigure 13: Auditory naming-related functional co-deactivation through each fasciculus.**

**eFigure 14: Visual naming-related functional co-deactivation through each fasciculus.**

**eFigure 15: Probability maps of stimulation-induced symptoms**

**eFigure 16: Association between auditory naming–related afferent flows and stimulation-induced manifestations.**

**eFigure 17: Association between visual naming–related afferent flows and stimulation-induced manifestations.**

**eFigure 1. Flowcharts showing the selection of patients who met eligibility criteria.** A total of 176 native English-speaking patients aged ≥4 years were enrolled. Of these, 24 did not complete the auditory naming task and 35 did not complete the visual naming task. An additional 14 patients were excluded due to major brain malformations, 7 due to a history of epilepsy surgery, and 10 due to right-hemispheric language dominance. Thus, intracranial EEG data were available from 121 patients for auditory naming and 110 patients for visual naming, with 104 patients contributing data for both tasks.

**eFigure 2. Intracranial electrode coverage. A** Number of artifact-free, non-epileptic sites analyzed for high-gamma activity during auditory (left) and picture naming (right). **B** Left hemispheric ROIs based on the FreeSurfer standard brain space; full names listed in **eTable 2**. The number of electrode sites and contributing patients for each ROI is also provided in **eTable 2**.

**eFigure 3**

**eFigure 3**

**eFigure 3**

**eFigure 3**

**eFigure 3**

**eFigure 3**

**eFigure 3**

**eFigure 3**

**eFigure 3**

**eFigure 3**

**eFigure 3**

**eFigure 3. Auditory naming-related high-gamma dynamics: means and 95% confidence intervals.** Mean high-gamma amplitude (% change relative to baseline) at each region of interest (ROI) is shown with 95% confidence intervals. Upper and lower horizontal bars indicate time bins with significant high-gamma augmentation and attenuation, respectively, relative to baseline; these bins were used to define functional coactivation and co-deactivation networks, as previously reported (Kochi et al., 2026). Red and blue bars denote time bins in which high-gamma amplitude was significantly greater or smaller than the mean of the immediately preceding ten bins for five consecutive bins; these were used to define accelerating and decelerating neural information flows. **A–F** Left hemispheric ROIs. **G–L** Right hemispheric ROIs. Numbers in parentheses indicate the number of patients who contributed data. For example, 19 patients contributed high-gamma amplitude data from the left anterior superior frontal gyrus.

**Supplementary reference:**

Kochi R, Kanno A, Uda H, et al. Whole-Brain Dynamic Causal Tractography Atlas of Auditory and Visual Speech Networks Derived From Intracranial Neurophysiologic Data. Neurology 2026;106:e214448.

**eFigure 4**

**eFigure 4**

**eFigure 4**

**eFigure 4**

**eFigure 4**

**eFigure 4**

**eFigure 4**

**eFigure 4**

**eFigure 4**

**eFigure 4**

**eFigure 4**

**eFigure 4. Visual naming-related high-gamma dynamics: means and 95% confidence intervals.** Mean high-gamma amplitude (% change relative to baseline) at each region of interest (ROI) is shown with 95% confidence intervals. Upper and lower horizontal bars indicate time bins with significant high-gamma augmentation and attenuation, respectively, relative to baseline; these bins were used to define functional coactivation and co-deactivation networks, as previously reported (Kochi et al., 2026). Red and blue bars denote time bins in which high-gamma amplitude was significantly greater or smaller than the mean of the immediately preceding ten bins for five consecutive bins; these were used to define accelerating and decelerating neural information flows. **A–F** Left hemispheric ROIs. **G–L** Right hemispheric ROIs. Numbers in parentheses indicate the number of patients who contributed data. For example, 21 patients contributed high-gamma amplitude data from the left anterior superior frontal gyrus.

**Supplementary reference:**

Kochi R, Kanno A, Uda H, et al. Whole-Brain Dynamic Causal Tractography Atlas of Auditory and Visual Speech Networks Derived From Intracranial Neurophysiologic Data. Neurology 2026;106:e214448.

**eFigure 5**

**eFigure 5**

**eFigure 5**

**eFigure 5**

**eFigure 5**

**eFigure 5**

**eFigure 5**

**eFigure 5**

**eFigure 5**

**eFigure 5**

**eFigure 5**

**eFigure 5. Auditory naming-related high-gamma amplitude modulations: leave-one-patient-out analysis.** Each plot shows the dynamics of high-gamma amplitude at each region of interest (ROI; % change relative to baseline) averaged across all patients except one excluded patient. **A–F** Left hemispheric ROIs. **G–L** Right hemispheric ROIs. To confirm that high-gamma amplitude modulations were robust and not disproportionately influenced by individual patients, we applied a leave-one-patient-out approach, computing Spearman’s correlation coefficient (rho) repeatedly while excluding each patient in turn. Across the 66 ROIs, the mean rho values ranged from 0.95 to 0.99. One-sample t-tests demonstrated that mean rho values were significantly greater than zero at all ROIs (all p < 0.00001). Numbers in parentheses indicate the number of patients who contributed data. For example, 19 patients contributed high-gamma amplitude data from the left anterior superior frontal gyrus.

**eFigure 6**

**eFigure 6**

**eFigure 6**

**eFigure 6**

**eFigure 6**

**eFigure 6**

**eFigure 6**

**eFigure 6**

**eFigure 6**

**eFigure 6**

**eFigure 6**

**eFigure 6. Visual naming-related high-gamma amplitude modulations: leave-one-patient-out analysis.** Each plot shows the dynamics of high-gamma amplitude at each region of interest (ROI; % change relative to baseline) averaged across all patients except one excluded patient. **A–F** Left hemispheric ROIs. **G–L** Right hemispheric ROIs. To confirm that high-gamma amplitude modulations were robust and not disproportionately influenced by individual patients, we applied a leave-one-patient-out approach, computing Spearman’s correlation coefficient (rho) repeatedly while excluding each patient in turn. Across the 66 ROIs, the mean rho values ranged from 0.96 to 0.99. One-sample t-tests demonstrated that mean rho values were significantly greater than zero at all ROIs (all p < 0.00001). Numbers in parentheses indicate the number of patients who contributed data. For example, 21 patients contributed high-gamma amplitude data from the left anterior superior frontal gyrus.

**eFigure 7**

**eFigure 7**

**eFigure 7**

**eFigure 7**

**eFigure 7. Auditory naming-related accelerating flows through each white matter fasciculus.** Bar height (Y-axis) represents the proportion of cortical ROI pairs connected by each white matter fasciculus that exhibited accelerating neural information flow within each time bin, as determined by transfer entropy–based effective connectivity. Bar color indicates the mean flow intensity. Numbers in parentheses indicate the number of cortical ROI pairs connected by each white matter fasciculus via the shortest structural pathway, according to the structural connectivity atlas of Kochi et al. (2026). For example, "Left arcuate fasciculus (n = 47)" indicates that the left arcuate fasciculus connected 47 cortical ROI pairs in the atlas; approximately 50% of these cortical ROI pairs exhibited accelerating neural information flow 100–200 ms before auditory stimulus offset.

**Supplementary reference:**

Kochi R, Kanno A, Uda H, et al. Whole-Brain Dynamic Causal Tractography Atlas of Auditory and Visual Speech Networks Derived From Intracranial Neurophysiologic Data. Neurology 2026;106:e214448.

**eFigure 8**

**eFigure 8**

**eFigure 8**

**eFigure 8**

**eFigure 8. Visual naming-related accelerating flows through each white matter fasciculus.** Bar height (Y-axis) represents the proportion of cortical ROI pairs connected by each white matter fasciculus that exhibited accelerating neural information flow within each time bin, as determined by transfer entropy–based effective connectivity. Bar color indicates the mean flow intensity. Numbers in parentheses indicate the number of cortical ROI pairs connected by each white matter fasciculus via the shortest structural pathway, according to the structural connectivity atlas of Kochi et al. (2026). For example, "Left arcuate fasciculus (n = 47)" indicates that the left arcuate fasciculus connected 47 cortical ROI pairs in the atlas; less than 20% of these cortical ROI pairs exhibited accelerating neural information flow 300–500 ms after visual stimulus offset.

**Supplementary reference:**

Kochi R, Kanno A, Uda H, et al. Whole-Brain Dynamic Causal Tractography Atlas of Auditory and Visual Speech Networks Derived From Intracranial Neurophysiologic Data. Neurology 2026;106:e214448.

**eFigure 9**

**eFigure 9**

**eFigure 9**

**eFigure 9**

**eFigure 9. Auditory naming-related decelerating flows through each white matter fasciculus.** Bar height (Y-axis) represents the proportion of cortical ROI pairs connected by each white matter fasciculus that exhibited decelerating neural information flow within each time bin, as determined by transfer entropy–based effective connectivity. Bar color indicates the mean flow intensity. Numbers in parentheses indicate the number of cortical ROI pairs connected by each white matter fasciculus via the shortest structural pathway, according to the structural connectivity atlas of Kochi et al. (2026). For example, "Left arcuate fasciculus (n = 47)" indicates that the left arcuate fasciculus connected 47 cortical ROI pairs in the atlas; less than 20% of these cortical ROI pairs exhibited decelerating neural information flow 300–500 ms before response onset as well as after response onset.

**Supplementary reference:**

Kochi R, Kanno A, Uda H, et al. Whole-Brain Dynamic Causal Tractography Atlas of Auditory and Visual Speech Networks Derived From Intracranial Neurophysiologic Data. Neurology 2026;106:e214448.

**eFigure 10**

**eFigure 10**

**eFigure 10**

**eFigure 10**

**eFigure 10. Visual naming-related decelerating flows through each white matter fasciculus.** Bar height (Y-axis) represents the proportion of cortical ROI pairs connected by each white matter fasciculus that exhibited decelerating neural information flow within each time bin, as determined by transfer entropy–based effective connectivity. Bar color indicates the mean flow intensity. Numbers in parentheses indicate the number of cortical ROI pairs connected by each white matter fasciculus via the shortest structural pathway, according to the structural connectivity atlas of Kochi et al. (2026). For example, "Left arcuate fasciculus (n = 47)" indicates that the left arcuate fasciculus connected 47 cortical ROI pairs in the atlas; less than 20% of these cortical ROI pairs exhibited decelerating neural information flow around response onset.

**Supplementary reference:**

Kochi R, Kanno A, Uda H, et al. Whole-Brain Dynamic Causal Tractography Atlas of Auditory and Visual Speech Networks Derived From Intracranial Neurophysiologic Data. Neurology 2026;106:e214448.

**eFigure 11**

**eFigure 11**

**eFigure 11**

**eFigure 11**

**eFigure 11. Auditory naming-related functional coactivation through each fasciculus.** Bar height represents the proportion of white matter pathways within each fasciculus exhibiting functional coactivation per time bin. Bar color indicates mean coactivation intensity. Functional coactivation networks were defined according to the methodology previously described in our iEEG study (Kochi et al., 2026).

**Supplementary reference:**

Kochi R, Kanno A, Uda H, et al. Whole-Brain Dynamic Causal Tractography Atlas of Auditory and Visual Speech Networks Derived From Intracranial Neurophysiologic Data. Neurology 2026;106:e214448.

**eFigure 12**

**eFigure 12**

**eFigure 12**

**eFigure 12**

**eFigure 12. Visual naming-related functional coactivation through each fasciculus.** Bar height represents the proportion of white matter pathways within each fasciculus exhibiting functional coactivation per time bin. Bar color indicates mean coactivation intensity. Functional coactivation networks were defined according to the methodology previously described in our iEEG study (Kochi et al., 2026).

**Supplementary reference:**

Kochi R, Kanno A, Uda H, et al. Whole-Brain Dynamic Causal Tractography Atlas of Auditory and Visual Speech Networks Derived From Intracranial Neurophysiologic Data. Neurology 2026;106:e214448.

**eFigure 13**

**eFigure 13**

**eFigure 13**

**eFigure 13**

**eFigure 13. Auditory naming-related functional co-deactivation through each fasciculus.** Bar height represents the proportion of white matter pathways within each fasciculus exhibiting functional co-deactivation per time bin. Bar color indicates mean co-deactivation intensity. Functional co-deactivation networks were defined according to the methodology previously described in our iEEG study (Kochi et al., 2026).

**Supplementary reference:**

Kochi R, Kanno A, Uda H, et al. Whole-Brain Dynamic Causal Tractography Atlas of Auditory and Visual Speech Networks Derived From Intracranial Neurophysiologic Data. Neurology 2026;106:e214448.

**eFigure 14**

**eFigure 14**

**eFigure 14**

**eFigure 14**

**eFigure 14. Visual naming-related functional co-deactivation through each fasciculus.** Bar height represents the proportion of white matter pathways within each fasciculus exhibiting functional co-deactivation per time bin. Bar color indicates mean co-deactivation intensity. Functional co-deactivation networks were defined according to the methodology previously described in our iEEG study (Kochi et al., 2026).

**Supplementary reference:**

Kochi R, Kanno A, Uda H, et al. Whole-Brain Dynamic Causal Tractography Atlas of Auditory and Visual Speech Networks Derived From Intracranial Neurophysiologic Data. Neurology 2026;106:e214448.

**eFigure 15**

**eFigure 15. Probability map of stimulation-induced symptoms.** **A** Auditory hallucinations. **B** Receptive aphasia. **C** Expressive aphasia. **D** Speech arrest. **E** Face sensorimotor symptoms. **F** Phosphene. **G** Visual distortion. **H** Picture naming error.

**eFigure 16. Association between auditory naming-related afferent flows and stimulation-induced manifestations.** Each plot shows Spearman’s rho, reflecting the correlation between mean accelerating flow intensity and the likelihood of stimulation-induced symptoms across time bins. **A** Correlation with auditory hallucinations. **B** Receptive aphasia. **C** Expressive aphasia. **D** Speech arrest. **E** Face sensorimotor symptoms. Horizontal bars mark intervals of significant correlation.

**eFigure 17. Association between visual naming-related afferent flows and stimulation-induced manifestations.** Each plot shows Spearman’s rho, reflecting the correlation between mean accelerating flow intensity and the likelihood of stimulation-induced symptoms across time bins. **A** Correlation with phosphene. **B** Visual distortion. **C** Picture naming error. **D** Speech arrest. **E** Face sensorimotor symptoms. Horizontal bars mark intervals of significant correlation.
