# Supplementary material for: Whole-brain millisecond-scale effective connectivity of auditory and visual naming
Source: Neuroimage. Author manuscript; Available in PMC 2026 Oct 13. (PMC13659335; doi:10.1016/j.neuroimage.2026.122236)
Supplement: Supplementary file 2 [file NIHMS2214451-supplement-2.docx]

**Supplementary Results: eResults**

***Effects of patient profiles on response times and neural dynamics***

As previously reported (Kochi et al., 2026), we used mixed-effects modeling to evaluate the effects of the following variables on response latency and high-gamma activity. We adopted this approach because the present study included 127 patients—two more than in Kochi et al. (2026).

Fixed effect predictors included the square root of age (√years), handedness, left-hemispheric epileptogenicity, presence of MRI-detectable lesions, and the number of antiseizure medications, with polytherapy used as an index of greater cognitive burdens. Median response time served as the dependent variable, with intercept and patient included as random effects. Analyses were performed in SPSS version 28 (IBM, Chicago, IL), and statistical significance was defined as two-sided p < 0.05. To evaluate neural responses, the same framework was applied to median high-gamma amplitudes across electrodes within each ROI, calculated in 100-ms bins from stimulus presentation through 500 ms after response, within a 2,500-ms analysis window. Significance thresholds were adjusted using Bonferroni correction (p < 0.05 across 66 ROIs and 25 time bins).

Increased √age was associated with shorter median response times in auditory (mixed model estimate: –0.20; 95%CI: –0.32 to –0.08; p-value: 0.001) and picture naming tasks (estimate: –0.19; 95%CI: –0.32 to –0.07; p-value: 0.003). A higher number of antiseizure medications was independently associated with a longer median response time in the auditory naming task (estimate: 0.14; 95%CI: 0.01 to 0.26; p-value: 0.03). These effects were independent of other fixed effect variables, none of which showed significant associations (p-values: 0.08–0.99).

Left-handedness was linked to a 15.1% increase in auditory naming–related high-gamma amplitude within the right entorhinal cortex (300–400 ms after stimulus offset; 95% CI: 9.9–20.3%; p = 7.8 × 10⁻⁶). Left-hemispheric epileptogenicity corresponded to a 29.6% increase in picture naming–related high-gamma amplitude at the right posterior superior temporal gyrus (0–100 ms before response onset; 95% CI: 17.7–41.5%; p = 6.6 × 10⁻⁶). No other fixed-effect variables were significantly associated with high-gamma amplitude.

**eReference:** Kochi R, Kanno A, Uda H, et al. Whole-Brain Dynamic Causal Tractography Atlas of Auditory and Visual Speech Networks Derived From Intracranial Neurophysiologic Data. Neurology 2026;106:e214448.
