# Supplementary material for: Whole-brain millisecond-scale effective connectivity of auditory and visual naming
Source: Neuroimage. Author manuscript; Available in PMC 2026 Oct 13. (PMC13659335; doi:10.1016/j.neuroimage.2026.122236)
Supplement: Supplementary file 3 [file NIHMS2214451-supplement-3.docx]

**Supplementary Tables: eTables**

**eTable1: Patient profiles.**

**eTable2: Number of artifact-free, nonepileptic electrode sites.**

| **Patient** |  | **MRI** | **Sampled hemisphere** | **Resected site** | **SOZ** | **Number of antiseizure medication** | **Median response time (seconds)** | |
| --- | --- | --- | --- | --- | --- | --- | --- | --- |
|  |  |  |  |  |  |  | **Auditory naming** | **Picture naming** |
| 1 |  | Tumor | Lt | Lt | Lt F | 3 | 0.751 | NA |
| 2 |  | Nonlesion | Rt | Rt | Rt O | 2 | 1.001 | NA |
| 3 |  | Tumor | Rt | Rt | NA | 1 | 0.827 | NA |
| 4 |  | Nonlesion | Lt | Lt | Lt FP | 1 | 1.353 | NA |
| 5 |  | Tumor | Lt | Lt | Lt T | 1 | 1.742 | NA |
| 6 |  | Tumor | Lt | Lt | NA | 1 | 0.668 | NA |
| 7 |  | Nonlesion | Lt and Rt | Rt | Rt FTPO | 3 | 1.450 | 1.425 |
| 8 |  | Nonlesion | Lt and Rt | Lt | Lt F | 1 | 0.883 | 1.067 |
| 9 |  | Dysplasia | Rt | Rt | Rt O | 3 | 0.949 | NA |
| 10 |  | Nonlesion | Rt | Rt | NA | 2 | 1.306 | NA |
| 11 |  | Tumor | Lt and Rt | Lt | NA | 1 | 2.158 | 2.771 |
| 12 |  | Nonlesion | Rt | Rt | Rt T | 2 | 1.339 | 1.798 |
| 13 |  | Nonlesion | Rt | Rt | Rt T | 1 | 1.799 | NA |
| 14 |  | Dysplasia | Rt | Rt | Rt PO | 3 | 0.664 | NA |
| 15 |  | Tumor | Lt | Lt | Lt T | 1 | 0.620 | 1.308 |
| 16 |  | Tumor | Lt | Lt | NA | 0 | 1.979 | NA |
| 17 |  | Nonlesion | Lt | Lt | NA | 2 | 0.684 | 1.583 |
| 18 |  | Nonlesion | Lt | Lt | Lt T | 1 | 2.285 | 2.694 |
| 19 |  | Tumor | Lt | Lt | Lt TP | 2 | 0.719 | 2.120 |
| 20 |  | Nonlesion | Lt and Rt | NA | Lt FT, Rt F | 2 | 1.265 | 1.031 |
| 21 |  | Tumor | Lt | Lt | Lt T | 2 | 1.155 | NA |
| 22 |  | Nonlesion | Lt and Rt | Rt | Rt P | 3 | 0.762 | NA |
| 23 |  | Nonlesion | Lt and Rt | Rt | NA | 2 | 1.459 | 1.441 |
| 24 |  | Nonlesion | Lt | Lt | Lt T | 3 | 1.285 | 1.283 |
| 25 |  | Dysplasia | Rt | Rt* | Rt FP | 2 | 0.808 | 1.237 |
| 26 |  | Tumor | Rt | Rt | Rt FP | 1 | 0.627 | 0.927 |
| 27 |  | Nonlesion | Lt and Rt | Lt | Lt F | 2 | 0.685 | 1.386 |
| 28 |  | Tumor | Rt | Rt | Rt T | 1 | 1.220 | 1.467 |
| 29 |  | Nonlesion | Lt | Lt | Lt F | 3 | 1.831 | 1.764 |
| 30 |  | Nonlesion | Lt and Rt | Lt | Lt PO | 2 | 0.904 | 0.816 |
| 31 |  | Nonlesion | Rt | Rt | Rt TPO | 2 | 0.983 | 1.327 |
| 32 |  | Tumor | Lt | Lt | NA | 1 | 0.832 | 0.925 |
| 33 |  | Nonlesion | Lt and Rt | Rt | Rt FP and Lt FP | 3 | 1.127 | 1.935 |
| 34 |  | Others | Lt | Lt | Lt FT | 5 | 2.178 | 1.825 |
| 35 |  | Nonlesion | Rt | Rt | Rt FP | 2 | 2.290 | 1.955 |
| 36 |  | Tumor | Lt | Lt | Lt O | 1 | 0.446 | 1.067 |
| 37 |  | Dysplasia | Rt | Rt | Rt TPO | 2 | 1.023 | 1.285 |
| 38 |  | Tumor | Lt | Lt | Lt T | 1 | 1.301 | 1.227 |
| 39 |  | Dysplasia | Rt | Rt | Rt TP | 2 | 1.121 | 1.157 |
| 40 |  | Dysplasia | Lt | Lt | Lt T | 2 | 1.499 | 1.255 |
| 41 |  | Dysplasia | Lt | Lt | NA | 3 | 1.099 | 1.042 |
| 42 |  | Nonlesion | Lt | Lt | Lt T | 2 | 2.563 | 3.064 |
| 43 |  | Nonlesion | Lt | Lt | Lt T | 2 | 1.015 | 1.187 |
| 44 |  | Others | Lt and Rt | Rt | Rt T | 2 | 1.425 | 1.188 |
| 45 |  | Nonlesion | Rt | Rt | NA | 3 | 2.958 | 2.306 |
| 46 |  | Nonlesion | Lt | Lt | NA | 2 | 0.736 | 2.087 |
| 47 |  | Nonlesion | Lt | Lt | Lt T | 3 | 2.289 | 1.813 |
| 48 |  | Nonlesion | Lt and Rt | Rt | Rt F | 1 | 1.329 | 2.272 |
| 49 |  | Dysplasia | Rt | Rt | Rt P | 3 | NA | 3.280 |
| 50 |  | Others | Rt | Rt | NA | 1 | 1.058 | 1.837 |
| 51 |  | Nonlesion | Rt | Rt | Rt T | 2 | 1.870 | 1.728 |
| 52 |  | Others | Lt | Lt | Lt P | 2 | 1.451 | 1.330 |
| 53 |  | Tumor | Rt | Rt | Rt T | 1 | 0.523 | 0.757 |
| 54 |  | Tumor | Lt and Rt | Lt | Lt F | 2 | 1.023 | 1.025 |
| 55 |  | Others | Rt | Rt | Rt T | 2 | 0.900 | 1.085 |
| 56 |  | Nonlesion | Lt and Rt | Rt | Rt T | 3 | 1.629 | 1.350 |
| 57 |  | Others | Lt and Rt | Lt | Lt T | 2 | 1.321 | 1.454 |
| 58 |  | Others | Lt | Lt | Lt T | 2 | 0.914 | 1.107 |
| 59 |  | Others | Lt | Lt | Lt T | 2 | 1.996 | 2.516 |
| 60 |  | Tumor | Lt | Lt | Lt T | 1 | 1.508 | 1.313 |
| 61 |  | Nonlesion | Lt and Rt | Rt | Rt T | 3 | 1.360 | 1.052 |
| 62 |  | Nonlesion | Lt | Lt | Lt T | 2 | 1.041 | 1.148 |
| 63 |  | Nonlesion | Rt | Rt | NA | 1 | 0.718 | 2.710 |
| 64 |  | Dysplasia | Lt | Lt | Lt P | 2 | NA | 1.099 |
| 65 |  | Tumor | Lt | Lt | Lt T | 2 | 1.337 | 1.030 |
| 66 |  | Nonlesion | Rt | Rt | Rt O | 2 | 0.544 | 0.763 |
| 67 |  | Nonlesion | Lt | Lt | Lt F | 2 | 1.016 | 1.078 |
| 68 |  | Dysplasia | Lt | Lt | Lt T | 1 | NA | 1.361 |
| 69 |  | Tumor | Rt | Rt | Rt TO | 1 | 2.273 | 2.508 |
| 70 |  | Nonlesion | Lt | Lt | Lt T | 1 | 1.167 | 1.052 |
| 71 |  | Nonlesion | Rt | Rt | Rt P | 2 | 1.164 | NA |
| 72 |  | Nonlesion | Lt | Lt | Lt T | 1 | 2.217 | 2.050 |
| 73 |  | Dysplasia | Rt | Rt | Rt T | 4 | 2.732 | 1.837 |
| 74 |  | Tumor | Lt | Lt | NA | 2 | 1.384 | 1.124 |
| 75 |  | Nonlesion | Rt | Rt | Rt F | 2 | 1.688 | 1.159 |
| 76 |  | Dysplasia | Lt | Lt | Lt T | 2 | 3.067 | 1.498 |
| 77 |  | Nonlesion | Rt | Rt | Rt P | 1 | 1.127 | 1.466 |
| 78 |  | Tumor | Rt | Rt | Rt P | 1 | 1.152 | 1.170 |
| 79 |  | Nonlesion | Lt | Lt | Lt F | 2 | 1.048 | 1.198 |
| 80 |  | Nonlesion | Rt | Rt | Rt F | 2 | 1.860 | 1.578 |
| 81 |  | Nonlesion | Lt | Lt | Lt T | 3 | 0.780 | 0.799 |
| 82 |  | Dysplasia | Rt | Rt | NA | 3 | 1.762 | 0.996 |
| 83 |  | Dysplasia | Rt | Rt | Rt TPO | 3 | 2.359 | NA |
| 84 |  | Dysplasia | Rt | Rt | Rt P | 1 | 1.964 | 3.123 |
| 85 |  | Dysplasia | Lt | Lt | Lt TO | 3 | 1.833 | 1.383 |
| 86 |  | Nonlesion | Lt and Rt | Lt | Lt T | 1 | 1.583 | 1.688 |
| 87 |  | Dysplasia | Lt | Lt | Lt F | 1 | NA | 1.692 |
| 88 |  | Nonlesion | Rt | Lt | Lt T | 2 | 1.129 | 2.076 |
| 89 |  | Nonlesion | Rt | Rt | Rt P | 1 | 0.987 | 1.286 |
| 90 |  | Others | Rt | Rt | Rt T | 2 | 1.068 | 0.761 |
| 91 |  | Nonlesion | Lt and Rt | NA | NA | 1 | 0.724 | 1.181 |
| 92 |  | Dysplasia | Rt | Rt | Rt T | 2 | 1.487 | 1.426 |
| 93 |  | Tumor | Lt | Lt | Lt T | 2 | 1.282 | 1.189 |
| 94 |  | Dysplasia | Rt | Rt | Rt T | 2 | 2.191 | 1.487 |
| 95 |  | Tumor | Lt and Rt | NA | Lt F | 2 | 1.292 | NA |
| 96 |  | Dysplasia | Rt | Rt | Rt O | 2 | 1.635 | 1.594 |
| 97 |  | Nonlesion | Lt | Lt | Lt PO | 2 | 1.332 | NA |
| 98 |  | Dysplasia | Lt | Lt | Lt TP | 1 | 1.120 | 1.649 |
| 99 |  | Nonlesion | Rt | Rt | NA | 2 | 1.807 | 1.633 |
| 100 |  | Nonlesion | Lt | Lt | Lt TPO | 3 | NA | 4.073 |
| 101 |  | Nonlesion | Rt | Rt | Rt F | 1 | 2.267 | 2.307 |
| 102 |  | Nonlesion | Lt and Rt | NA | Lt F | 1 | 1.919 | 1.476 |
| 103 |  | Nonlesion | Rt | Rt | Rt P | 2 | 1.497 | 1.260 |
| 104 |  | Tumor | Rt | Rt | Rt I | 2 | 1.729 | 1.210 |
| 105 |  | Dysplasia | Lt and Rt | Rt | Rt F | 1 | 2.338 | 2.016 |
| 106 |  | Nonlesion | Rt | NA | NA | 2 | 1.363 | 1.207 |
| 107 |  | Dysplasia | Lt | Lt | Lt O | 3 | 1.563 | 0.761 |
| 108 |  | Nonlesion | Lt and Rt | Rt | Rt F | 2 | 0.915 | 1.815 |
| 109 |  | Tumor | Lt | Lt | Lt T | 1 | 2.304 | 1.272 |
| 110 |  | Nonlesion | Lt and Rt | Rt | Rt F | 2 | 1.078 | 0.861 |
| 111 |  | Dysplasia | Lt | Lt | Lt TO | 5 | 2.339 | 1.343 |
| 112 |  | Nonlesion | Rt | Rt | Rt TO | 3 | 2.279 | 1.282 |
| 113 |  | Nonlesion | Rt | Rt | Rt F | 2 | 1.332 | 1.140 |
| 114 |  | Tumor | Lt and Rt | Lt | Lt TP | 2 | 1.435 | 1.385 |
| 115 |  | Dysplasia | Lt and Rt | Lt | Lt F | 2 | 1.283 | 1.017 |
| 116 |  | Nonlesion | Lt and Rt | Rt | Rt TPO and Lt TO | 2 | 1.577 | 1.951 |
| 117 |  | Dysplasia | Lt and Rt | Rt | Rt P | 3 | 2.196 | 0.909 |
| 118 |  | Tumor | Lt | Lt | NA | 1 | 2.867 | 2.420 |
| 119 |  | Tumor | Lt | Lt | NA | 3 | 1.384 | 1.036 |
| 120 |  | Nonlesion | Lt and Rt | Rt | Rt F | 3 | 1.994 | 1.282 |
| 121 |  | Tumor | Rt | Rt | NA | 2 | 2.195 | 1.514 |
| 122 |  | Dysplasia | Lt and Rt | Lt | Lt F | 2 | NA | 2.626 |
| 123 |  | Nonlesion | Lt | Lt** | NA | 2 | 1.162 | 0.859 |
| 124 |  | Others | Lt | Lt | Lt T | 3 | 2.667 | 1.425 |
| 125 |  | Others | Lt and Rt | Rt | Rt TPO | 2 | 1.767 | 1.703 |
| 126 |  | Dysplasia | Lt | Lt | Lt P | 2 | 1.910 | 1.223 |
| 127 |  | Nonlesion | Rt | Rt | Rt T | 3 | 2.361 | 1.256 |

**eTable 1. Patient profiles.** Sixty-one females (F), and 66 males (M) were included in the study. Patient age distribution: 24 patients aged 5–9 years, 48 aged 10–14 years, 41 aged 15–19 years, and 14 aged 20 years or older. Handedness: 117 right-handed, 9 left-handed, and 1 ambidextrous. Lt: left. Rt: right. SOZ: seizure onset zone. NA: not available. MRI lesions other than dysplasia and tumor included focal cortical atrophy (patient 34, 52, 125), hippocampal sclerosis (patients 44, 57, 58, 59, 90, 124), arachnoid cyst (patient 50), and arteriovenous malformations (patient 55). *: multiple subpial transections on the right Rolandic areas. **: responsive neurostimulation employed to the left temporal region. F: Frontal. T: Temporal. O: Occipital. P: Parietal. I: insula.

| **Auditory naming** | | |  | **Picture naming** | | |
| --- | --- | --- | --- | --- | --- | --- |
| **Regions of interest** | **Left** | **Right** |  | **Regions of interest** | **Left** | **Right** |
| aSFG: anterior superior frontal gyrus | 82 (19) | 143 (35) |  | aSFG: anterior superior frontal gyrus | 91 (21) | 136 (32) |
| pSFG: posterior superior frontal gyrus | 79 (23) | 99 (35) |  | pSFG: posterior superior frontal gyrus | 85 (25) | 88 (30) |
| rMFG: rostral middle frontal gyrus | 254 (47) | 339 (51) |  | rMFG: rostral middle frontal gyrus | 259 (43) | 296 (46) |
| cMFG: caudal middle frontal gyrus | 230 (52) | 259 (54) |  | cMFG: caudal middle frontal gyrus | 226 (48) | 229 (48) |
| pOp: pars opercularis | 158 (47) | 107 (45) |  | pOp: pars opercularis | 152 (43) | 96 (37) |
| pTr: pars triangularis | 110 (38) | 180 (44) |  | pTr: pars triangularis | 108 (41) | 156 (38) |
| pOrb: pars orbitalis | 83 (32) | 83 (26) |  | pOrb: pars orbitalis | 82 (31) | 72 (24) |
| LOrbF: lateral orbitofrontal gyrus | 120 (38) | 99 (33) |  | LOrbF: lateral orbitofrontal gyrus | 116 (36) | 97 (30) |
| MOrbF: medial orbitofrontal gyrus | 27 (19) | 40 (24) |  | MOrbF: medial orbitofrontal gyrus | 28 (20) | 39 (24) |
| aCG: anterior cingulate gyrus | 13 (8) | 18 (9) |  | aCG: anterior cingulate gyrus | 13 (8) | 17 (9) |
| IG: insular gyrus | 4 (2) | 4 (2) |  | IG: insular gyrus | 6 (3) | 6 (3) |
| aSTG: anterior superior temporal gyrus | 162 (47) | 224 (50) |  | aSTG: anterior superior temporal gyrus | 172 (46) | 185 (42) |
| pSTG: posterior superior temporal gyrus | 271 (54) | 207 (49) |  | pSTG: posterior superior temporal gyrus | 254 (50) | 158 (40) |
| aMTG: anterior middle temporal gyrus | 91 (32) | 89 (32) |  | aMTG: anterior middle temporal gyrus | 101 (32) | 85 (32) |
| pMTG: posterior middle temporal gyrus | 258 (50) | 131 (39) |  | pMTG: posterior middle temporal gyrus | 231 (46) | 88 (31) |
| aITG: anterior inferior temporal gyrus | 65 (26) | 99 (35) |  | aITG: anterior inferior temporal gyrus | 65 (25) | 81 (30) |
| pITG: posterior inferior temporal gyrus | 168 (52) | 105 (41) |  | pITG: posterior inferior temporal gyrus | 174 (49) | 91 (37) |
| aFG: anterior fusiform gyrus | 44 (24) | 76 (36) |  | aFG: anterior fusiform gyrus | 47 (24) | 63 (29) |
| pFG: posterior fusiform gyrus | 198 (47) | 161 (47) |  | pFG: posterior fusiform gyrus | 201 (47) | 153 (42) |
| EG: entorhinal gyrus | 48 (23) | 69 (28) |  | EG: entorhinal gyrus | 49 (24) | 60 (23) |
| PHG: parahippocampal gyrus | 38 (19) | 27 (20) |  | PHG: parahippocampal gyrus | 37 (18) | 24 (17) |
| sPreCG: superior precentral gyrus | 113 (40) | 127 (42) |  | sPreCG: superior precentral gyrus | 115 (42) | 105 (38) |
| iPreCG: inferior precentral gyrus | 362 (59) | 404 (57) |  | iPreCG: inferior precentral gyrus | 367 (57) | 359 (49) |
| sPoCG: superior postcentral gyrus | 119 (41) | 91 (41) |  | sPoCG: superior postcentral gyrus | 113 (38) | 88 (35) |
| iPoCG: inferior postcentral gyrus | 328 (59) | 321 (55) |  | iPoCG: inferior postcentral gyrus | 335 (56) | 282 (49) |
| PCL: paracentral lobule | 32 (15) | 65 (24) |  | PCL: paracentral lobule | 42 (18) | 54 (21) |
| SPG: superior parietal gyrus | 69 (19) | 57 (19) |  | SPG: superior parietal gyrus | 65 (18) | 33 (14) |
| IPG: inferior parietal gyrus | 96 (26) | 162 (38) |  | IPG: inferior parietal gyrus | 91 (26) | 129 (34) |
| SMG: supramarginal gyrus | 353 (56) | 339 (51) |  | SMG: supramarginal gyrus | 343 (53) | 290 (46) |
| LOG: lateral occipital gyrus | 229 (41) | 217 (42) |  | LOG: lateral occipital gyrus | 244 (41) | 205 (38) |
| LG: lingual gyrus | 122 (34) | 171 (46) |  | LG: lingual gyrus | 132 (36) | 160 (42) |
| CG: cuneus gyrus | 23 (12) | 39 (21) |  | CG: cuneus gyrus | 26 (15) | 35 (18) |
| Pcun: precuneus gyrus | 52 (23) | 75 (29) |  | Pcun: precuneus gyrus | 49 (24) | 66 (25) |
| pCG: posterior cingulate gyrus | 67 (22) | 83 (27) |  | pCG: posterior cingulate gyrus | 67 (23) | 73 (25) |
| Total | 4468 (73) | 4710 (72) |  | Total | 4486 (70) | 4099 (63) |

**eTable 2. Number of artifact-free, nonepileptic electrode sites.** Numbers in parentheses indicate the number of patients who contributed data. The IG region was excluded from the group-level region-of-interest analysis because fewer than seven patients in a hemisphere provided usable data.
