# Supplementary material for: Whole-brain millisecond-scale effective connectivity of auditory and visual naming
Source: Neuroimage. Author manuscript; Available in PMC 2026 Oct 13. (PMC13659335; doi:10.1016/j.neuroimage.2026.122236)
Supplement: Supplementary file 4 [file NIHMS2214451-supplement-4.docx]

**Legends for Supplementary Videos: eVideo Legends**

**eVideo 1. Neural information flows and functional coactivation/co-deactivation during auditory naming.** Left: Accelerating (red/yellow) and decelerating (blue/green) flows through white matter tracts. Right: Functional coactivation (red/yellow) and co-deactivation (blue/green).

**eVideo 2. Neural information flows and functional coactivation/co-deactivation during visual naming.** Left: Accelerating (red/yellow) and decelerating (blue/green) flows through white matter tracts. Right: Functional coactivation (red/yellow) and co-deactivation (blue/green).

**eVideo 3. Neural information flows and cortical high-gamma modulations during auditory naming.** Left: Accelerating (red/yellow) and decelerating (blue/green) flows through white matter tracts. Right: Cortical high-gamma modulations.

**eVideo 4. Neural information flows and cortical high-gamma modulations during visual naming.** Left: Accelerating (red/yellow) and decelerating (blue/green) flows through white matter tracts. Right: Cortical high-gamma modulations.
